# FEZ2 Has Acquired Additional Protein Interaction Partners Relative to FEZ1: Functional and Evolutionary Implications

**DOI:** 10.1371/journal.pone.0017426

**Published:** 2011-03-08

**Authors:** Marcos R. Alborghetti, Ariane S. Furlan, Jörg Kobarg

**Affiliations:** 1 Laboratório Nacional de Biociências, Centro Nacional de Pesquisa em Energia e Materiais, Campinas, São Paulo, Brasil; 2 Departamento de Bioquímica-Programa de Pós-graduação em Biologia Funcional e Molecular, Instituto de Biologia, Universidade Estadual de Campinas, Campinas, São Paulo, Brasil; University College Dublin, Ireland

## Abstract

**Background:**

The FEZ (fasciculation and elongation protein zeta) family designation was purposed by Bloom and Horvitz by genetic analysis of *C. elegans unc-76*. Similar human sequences were identified in the expressed sequence tag database as *FEZ1* and *FEZ2*. The *unc-76* function is necessary for normal axon fasciculation and is required for axon-axon interactions. Indeed, the loss of UNC-76 function results in defects in axonal transport. The human FEZ1 protein has been shown to rescue defects caused by *unc-76* mutations in nematodes, indicating that both UNC-76 and FEZ1 are evolutionarily conserved in their function. Until today, little is known about FEZ2 protein function.

**Methodology/Principal Findings:**

Using the yeast two-hybrid system we demonstrate here conserved evolutionary features among orthologs and non-conserved features between paralogs of the FEZ family of proteins, by comparing the interactome profiles of the C-terminals of human FEZ1, FEZ2 and UNC-76 from *C. elegans*. Furthermore, we correlate our data with an analysis of the molecular evolution of the FEZ protein family in the animal kingdom.

**Conclusions/Significance:**

We found that FEZ2 interacted with 59 proteins and that of these only 40 interacted with FEZ1. Of the 40 FEZ1 interacting proteins, 36 (90%), also interacted with UNC-76 and none of the 19 FEZ2 specific proteins interacted with FEZ1 or UNC-76. This together with the duplication of unc-76 gene in the ancestral line of chordates suggests that FEZ2 is in the process of acquiring new additional functions. The results provide also an explanation for the dramatic difference between C. elegans and D. melanogaster unc-76 mutants on one hand, which cause serious defects in the nervous system, and the mouse FEZ1 -/- knockout mice on the other, which show no morphological and no strong behavioural phenotype. Likely, the ubiquitously expressed FEZ2 can completely compensate the lack of neuronal FEZ1, since it can interact with all FEZ1 interacting proteins and additional 19 proteins.

## Introduction

The FEZ (fasciculation and elongation protein zeta) family designation was first purposed by Bloom and colleagues in 1997 by genetic analysis of *C. elegans unc-76*. The 376 and 385 amino acid containing isoforms of the UNC-76 protein arise by alternative splicing of the same gene and showed no strong similarity to any previously characterized proteins, but similar human sequences were identified in the expressed sequence tag database, dbEST [1], [2] and Bloom & Horvitz named the two identified human genes *FEZ1* and *FEZ2*.

The *unc-76* function is necessary for normal fascicle structure and is required specifically for axon-axon interactions in *C. elegans*. The human *FEZ1* gene was able to restore partial locomotion and axonal fasciculation in the *C. elegans unc-76* mutants in germ-line transformation experiments, indicating that both the function and the structure of the FEZ proteins have been conserved in evolution [1]. Loss of *Drosophila Unc-76* function results in locomotion and axonal transport defects reminiscent of phenotypes observed in kinesin mutants and thereby suggesting that UNC-76 is required for kinesin-dependent axonal transport [3]. The FEZ1-deficient mice however, did not exhibit any obvious abnormal brain architecture, although they manifest slight behavioral abnormalities, including a hyperlocomotion phenotype and enhanced responsiveness to psychostimulants [4].

The homologous proteins UNC-76, FEZ1 and FEZ2, share a conserved predicted coiled-coil region at their C-terminal regions [1], [5], [6]. Coiled-coils are autonomous folding units consisting of two to four α-helices that wrap around each other with a slight left-handed super-helical twist [7] and thereby mediate sub-unit oligomerization [8] as well as protein-protein interactions [9]. Up to now 4 interactions were reported for *C. elegans* UNC-76, 45 interactions for human FEZ1 and 17 interactions for human FEZ2 according to BIND, HPRD, BioGRID data and previous research published by our group [10]. All of these interactions occur at the C-terminus with the predicted coiled-coil region. Some of these interactions implicate FEZ1 protein in the context of schizophrenia (DISC1) [11], [12], aspects of viral infection such as post-entry block of retroviral infection [13] and inhibition of the release of progeny virions (agnoprotein of the human polyomavirus JC virus) [14], and naturally in neuritogenesis (NBR1, PKCζ, DISC1) [5], [11], [15]. Furthermore, the association between FEZ1 and E4B was enhanced by co-expression of a constitutively active form of protein kinase Cζ, and phosphorylation of FEZ1 by this kinase and its subsequent ubiquitylation by E4B resulted in neurite extension in PC12 cells [4], [16]. FEZ1 also contributes to the polarization of hippocampal neurons by controlling mitochondrial motility [4], [17].

Here, we report that nucleotide sequences belonging to the FEZ family can be found in many eukaryotic species, but that most of the putative homologs are not only functionally uncharacterized but even remain unrecognized as FEZ family members in the databases. Our phylogenetic analysis of the FEZ family of proteins indicates that the ancestral gene of the FEZ family is found only in the animal kingdom. The gene duplication of *unc-76* to *FEZ1* and *FEZ2* is predicted to have occurred during the two rounds of whole-genome duplication in the chordate ancestral line, after the divergence of cephalochordates but before the splitting of the teleosts and tetrapods taxa.

The most conserved region in FEZ proteins is its C-terminus, and we and others showed previously that this region is involved in the association with other proteins [10], [18]. Here, yeast two-hybrid assays with *C. elegans* UNC-76 (248–372), human FEZ1 (221–392) and human FEZ2 (207–353) as baits and human prey clones from cDNA libraries demonstrated that the pattern of protein-protein interactions (PPIs) is highly conserved between UNC-76 and FEZ1. These orthologues did however not interact with 19 proteins that interacted specifically with FEZ2, although FEZ2 interacted with all 40 proteins that interact with FEZ1/UNC-76. In summary, we found largely overlapping PPI patterns for FEZ1 and UNC-76 and extended PPIs for FEZ2. Interestingly, our data provide an explanation for the ability of *FEZ1* to rescue the defects caused by *unc-76* mutations in nematodes and to the lack of strong defects in *FEZ1* deficient mice, since the continuous presence of the ubiquitous expressed paralogue FEZ2 may be able to compensate lack of FEZ1 because its PPI pattern contains all the interactions found for FEZ1.

## Methods

### Protein sequence analysis and multiple sequence alignments

**Figure 1:**
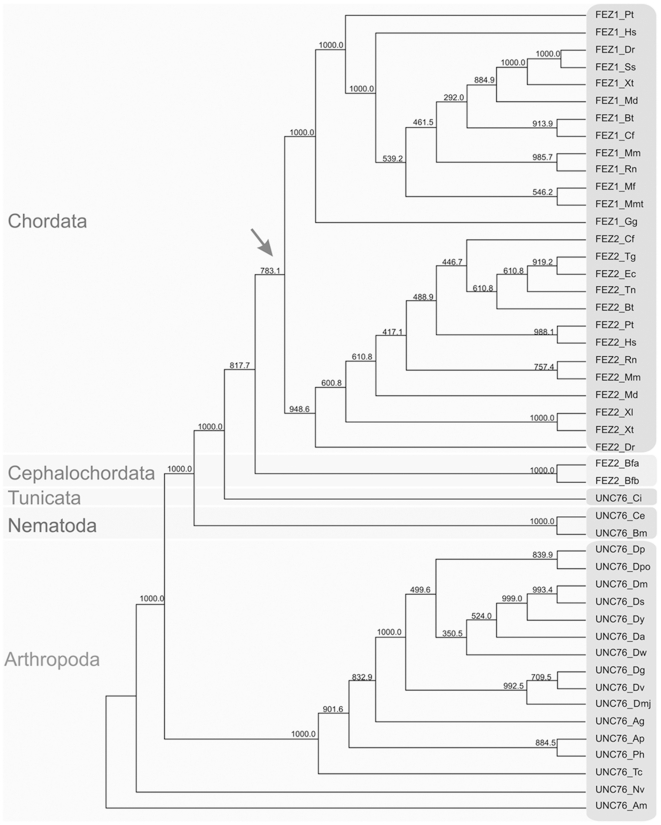
A phylogenetic tree of the FEZ protein family. The tree was derived by the parsimony method (Phylip protpars). The bootstrap multidataset and resampling method was employed and the numbers on the branches indicate the number of times the partition of the species into the two sets which are separated by that branch occurred among the trees out of 999.96 trees. The arrow indicates the probable point of FEZ gene duplication. Ag – *Anopheles gambiae*, Am – *Apis mellifera*, Ap – *Acyrthosiphon pisum*, Bf – *Branchiostoma floridae* (Bfa and Bfb refer to two sequences [polymorphisms] of selected hypothetical genes in this species), Bm – *Brugia malayi*, Bt – *Bos taurus*, Ce – *Caenorhabditis elegans*, Cf – *Canis familiaris*, Ci – *Ciona intestinalis*, Da – *Drosophila ananassae*, Dg – *Drosophila grimshawi*, Dm – *Drosophila melanogaster*, Dmj – *Drosophila mojavensis*, Dp – *Drosophila persimilis*, Dpo – *Drosophila pseudoobscura*, Dr – *Danio rerio*, Ds – *Drosophila sechellia*, Dv – *Drosophila virilis*, Dw – *Drosophila willistoni*, Dy – *Drosophila yakuba*, Ec – *Equus caballus*, Gg –*Gallus gallus*, Hs – *Homo sapiens*, Md – *Monodelphis domestica*, Mf – *Macaca fascicularis*, Mm – *Mus musculus*, Mmt – *Macaca mulatta*, Nv – *Nasonia vitripennis*, Ph – *Pediculus humanus*, Pt – *Pan troglodytes*, Rn – *Rattus norvegicus*, Ss – *Salmo salar*, Tc – *Tribolium castaneum*, Tg –*Taeniopygia guttata*, Tn – *Tetraodon nigroviridis*, Xl – *Xenopus laevis*, Xt – *Xenopus tropicalis*.

Protein Psi-Blast [19] searches with the full length *C. elegans* UNC-76 sequence were performed at the NCBI Web site http://www.ncbi.nlm.nih.gov/BLAST/ using the non-redundant protein sequence database available at August 16, 2009. After six rounds of iteration, all UNC-76, FEZ1 and FEZ2 orthologs with an E-value of 0.005 or below were selected and all redundant sequences were excluded (except to *B. floridae*). All sequences were collected in FASTA format for further analysis as shown in Table S1. The identification and posterior naming of the protein sequences as either UNC-76, FEZ1 or FEZ2 is based on the phylogenetical analyses shown in Figure 1 and Figure S1. The sequences were aligned using the ClustalW2 Web Site http://www.ebi.ac.uk/Tools/clustalw2/index.html at default settings. The alignments were then shaded using the multiple sequence alignment editor GENEDOC http://www.nrbsc.org/gfx/genedoc/index.html. The identity and similarity were calculated using default settings by NPS@: Network Protein Sequence Analysis trough the alignments of human proteins FEZ1 and FEZ2 and *C. elegans* UNC-76 protein (http://npsa-pbil.ibcp.fr/cgi-bin/align_clustalw.pl) [20].

### Phylogenetical analyses

PHYLIP version 3.5c [21] was used for the phylogenetical analyses at the web site http://mobyle.pasteur.fr/cgi-bin/portal.py?jobs=http://mobyle.pasteur.fr/data/jobs/protpars. Parsimony analyses were performed using the protein alignment as input. Bootstrap values were obtained by using SEQBOOT and creating 1000 Bootstrap data sets. Analysis was then performed using PROTPARS with standard parameters. The “M” option was invoked for the analysis of the multiple data sets generated by SEQBOOT.

### Plasmid constructions

The plasmid constructions of pBMT116-FEZ1 (221–392) (NM_005103.3) and pBTM-FEZ2 (207–353) (NM_005102.2) have been described previously [10]. The nucleotide sequence coding residues 248–372 of *C. elegans* UNC-76 (NM_074311.3) was PCR-amplified using a specific primer set (5′- GAATTCGATAATCTTCAAGAGCTCTCC-3′ , 5′- GTCGACCTAACACGATATATTTTTTGG-3′) as well as the template plasmid pSU001, that had been generously provided by Dr. Hengartner [4]. Subsequently the fragment was cloned in pGEM®-T vector (Promega), and subcloned via *Eco*RI and *Sal*I restriction sites into the vector pBTM116. The orientation, frame, and correctness of sequence of each insert DNA was confirmed by restriction endonuclease analysis and automated DNA sequencing. These are no new cell lines but only cDNA clones obtained by *in vitro* experiments as described.

### Yeast two-hybrid screen and DNA sequence analysis

**Figure 2:**
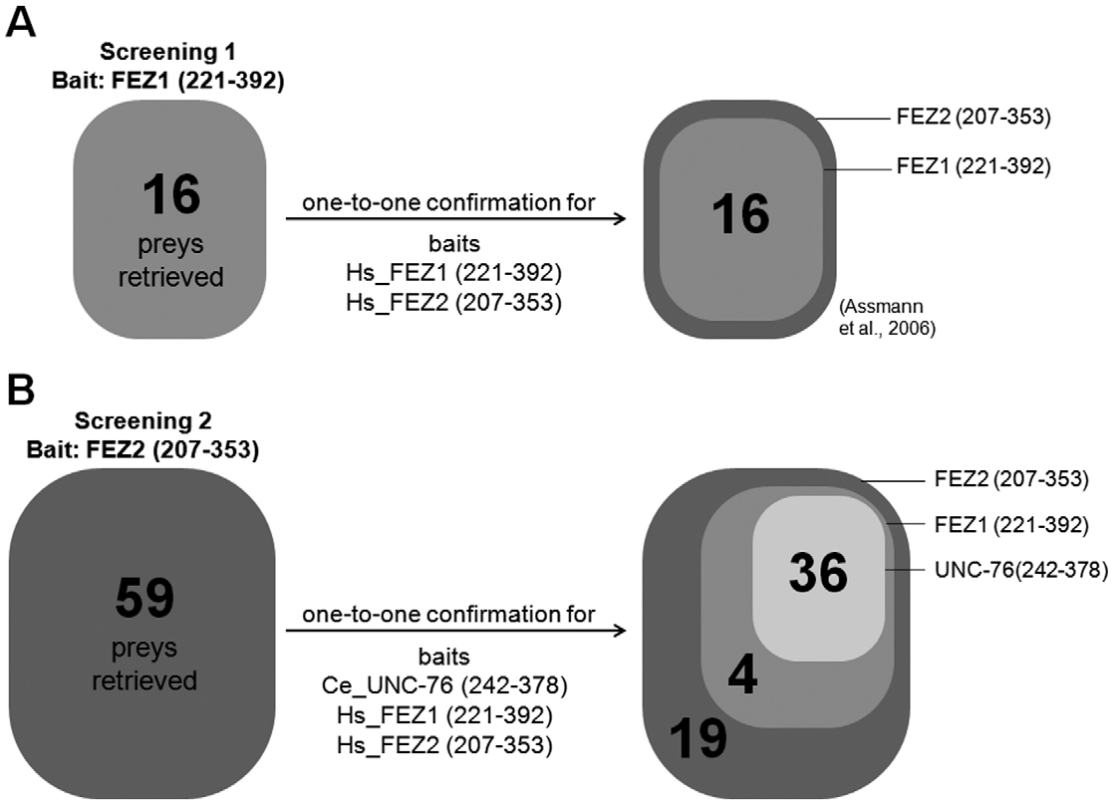
Schematic representation summarizing the primary yeast two-hybrid screens with UNC-76, FEZ1 and FEZ2 as baits. A) Yeast two-hybrid screen performed with FEZ1 (221–392) as a bait using a human fetal brain cDNA library retrieved 16 proteins that were confirmed to interact all with both FEZ1(221–392) and FEZ2 (207–353) in the one-to-one assay. B) Yeast two-hybrid screen performed with FEZ2 (207–353) as a bait using human fetal brain and bone marrow cDNA libraries retrieved 59 proteins as preys. In the one-to-one confirmation, 19 of these proteins interacted only with FEZ2 (207–353) protein, 40 with both FEZ2 (207–353) and FEZ1 (221–392) and 36 interacted with all FEZ2 (207–353), FEZ1 (221–392) and UNC-76 (242–378).

Using FEZ1 as a bait in a screening, Assmann et al. (2006) [10] identified 16 proteins interacting with FEZ1 and all of them interacted with FEZ2 protein (Fig. 2A). In this work, yeast two-hybrid screens [22] of human fetal brain and bone marrow cDNA libraries (Clontech) were performed as described previously by using the yeast strain L40 (trp1-901, his3Δ200, leu2-3, ade2 LYS2::(lexAop)4-HIS3 URA3::(lexAop)8-lac GAL4) and human FEZ2 (207–353) as a bait in fusion with the LexA protein as encoded in the recombinant vector pBTM116 [23]. This fragment of FEZ2 does not auto-activate the yeast reporter genes (as FEZ proteins full length), neither did so the C-terminal constructs of FEZ1 and UNC-76 used in one-to-one confirmation. The autonomous activation test for HIS3 was performed in minimal medium plates without tryptophane and histidine but containing 10, 20, 30, or 50 mM of 3-amino-1,2,4-triazole (3-AT). Furthermore, the autonomous activation of LacZ was measured by the beta-galactosidase filter assay as described below. Yeast cells were transformed according to the protocols supplied by the cDNA library manufacturer (Clontech). The screening with FEZ2 (207–353) as bait was performed in minimal medium plates without tryptophan, leucine, and histidine and with addition of 10 mM of 3-AT to repress unspecific background growth.Recombinant pACT2 plasmids of positive clones were isolated and their insert DNAs were sequenced with a DNA sequencer model 377S (Applied Biosystems, Foster City, CA). The obtained DNA sequence data were compared with sequences in the NCBI data bank using the BLASTX 2.2.12 program [19]. The corresponding Accession numbers of the DNA sequences identified are given in the Table S1. As no new sequences have been obtained no new sequence data have been deposited in the GenBank. After identification, one representative clone of each prey protein was selected to yeast two-hybrid one-to-one confirmation.

### Yeast Two-hybrid assays one-to-one confirmation with the homologues FEZ1, FEZ2 and UNC-76

Yeast two-hybrid assays were performed with human FEZ1 (221–392) and *C. elegans* UNC-76 (248–372) as additional baits fused to LexA in vector pBTM116, as described above and previously [10]. The recombinant pACT2 plasmids of positives clones isolated from the library screens with bait FEZ2 (207–353) were used as preys and the interactions with FEZ1 and UNC-76 (and control FEZ2) were analyzed by growth in minimal medium plates and activation of the LacZ using the standard beta-galactosidase filter assay described in the following section (Fig. 2B).

### Assay for beta-galactosidase activity in yeast cells

beta-Galactosidase activity in yeast cells was determined by the filter assay method. Yeast transformants (Leu^+^, Trp^+^, and His^+^) were transferred onto nylon membranes, permeabilized in liquid nitrogen, and placed on Whatman 3 MM paper previously soaked in Z buffer (60 mM Na_2_HPO_4_, 40 mM NaH_2_PO_4_, 10 mM MgCl_2_, 50 mM 2-mercaptoethanol, pH 7.0) containing 1 mg/ml 5-bromo-4-chloro-3-indolyl-β-D-galactoside (X-gal). After incubation at 37°C for 1 h, the yeast cells forming dark blue colonies were taken from replica plates for further analysis (primary screen).

## Results

### Identification of members of the FEZ family and of conserved regions in their amino acid sequences

A database of sequences judged to be members of the FEZ protein family was compiled (Table S1, see Material and Methods section). Altogether 47 members for the FEZ family were identified by Psi-Blast searches with the *C. elegans* UNC-76 sequence in the non-redundant protein sequence database. FEZ1 family sequences were found in a variety of species ranging from Nematodes, via Arthropods to Mammals but not in Plants, Fungi or Protists. Multiple Clustal W alignments of sequences of the FEZ family identified a highly conserved region both at the N-terminus (ca. aa 160–180 in FEZ1) and at the C-terminus (Figure S1). The latter consists mainly in coiled-coil regions [10], [24], [25] located at the C-terminus which mediate the majority of the protein-protein interactions (ca. aa 230–307 in FEZ1) [10]. The alignment revealed, that the nuclear localization signal (NLS) (KKRRK, ca. aa 290–294 in FEZ1) [26], which consists of basic amino acids, is quite conserved in all FEZ1 genes that resulted from the putative *unc-76* gene duplication (see details below), suggesting that new nuclear functions may have been acquired in FEZ1 afterwards. Although there is no striking NLS in FEZ2 sequences, they contain a conserved polybasic region (e.g. KKKKK) with conserved amino acid substitutions in the FEZ1 related NLS. The NLS is less well conserved in all UNC-76 sequences.

### Phylogenetic analysis of the FEZ gene duplication

A phylogenetic tree (Fig. 1) was generated from the Clustal W alignment (Figure S1) of the all identified FEZ protein sequences (Table S1) using the parsimony method. Bootstrap options to resampling methods were defined to contain 1000 replications. The presence of FEZ1 and FEZ2 in all analyzed Actinopterygii (ray-finned fishes) genomes suggests that the *FEZ1* and *FEZ2* genes originated from the ancestral *unc-76* by gene or whole genome duplications before the radiation of the Actinopterygii but after the divergence of the amphioxus branch and hence concomitant origin of the chordata. In fact, according to an amphioxus-human synteny analysis by Putnam [27] two rounds of whole-genome duplication seem to have occurred in the chordate stemline, after the divergence of the cephalochordates but before the split into teleosts and tetrapods (Fig. 1). Hence, the *unc-76* gene seems to have duplicated in the chordate stemline into *FEZ1* and *FEZ2* genes.

### Identification of proteins that interact with FEZ2

So far, only interactions for C-terminal of FEZ proteins are described, as by our screenings using C termini of FEZ proteins (due to auto-activation of full length and N-terminal constructs of the FEZ proteins as baits in these assays), as well as by other publications describing FEZ proteins recovered as prey in other screens (in this case, the interaction region was mapped to be the C-terminal region) [5], [14]. In a previous study, we had identified proteins that interact with human FEZ1 using its C-terminal region (221–392) as a bait in a yeast two-hybrid screen of a human fetal brain cDNA library [10]. We further found that all of the 16 interacting proteins identified were also able to interact with FEZ2 (207–353). The interacting proteins are functionally involved in transcriptional regulation (6 proteins), neuronal cell development (2 proteins), intracellular transport processes (3 proteins), apoptosis (2 proteins), or were of unknown function (3 proteins). Although these data suggested that FEZ1 and FEZ2 have largely overlapping protein interaction profiles we wanted to deepen our insights by performing another two-hybrid screen with the human paralogue FEZ2 as well as by introducing an evolutionary component by comparing the interaction profiles of both proteins with the orthologous *C. elegans* protein UNC-76, which may have preserved ancient features.

**Figure 3:**
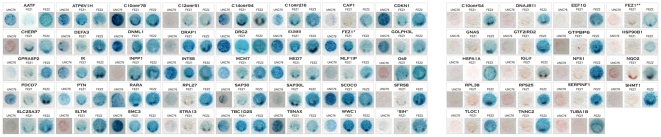
Protein-protein interaction pattern among homologs by beta-galactosidase assay. Result of the yeast two-hybrid assay with the C-terminal of the three homologous proteins UNC-76 (242–378), FEZ1 (221–392) and FEZ2 (207–353) as baits. All 59 clones obtained here by our yeast two-hybrid library screens with the C-terminal of FEZ2 (207–353) were tested against all three bait constructs one-to-one. The pattern of interaction between the orthologous FEZ1 and UNC-76 was almost identical, indicating a conservation of the UNC-76 function in human FEZ1. The clones that showed specific interactions with FEZ2 only, are separated on the right side of the figure. The tests were performed in duplicate, but only one representative result is shown.

To this end we started by employing the yeast two-hybrid system [23] to screen humans fetal brain and bone marrow cDNA libraries. The truncated FEZ2 (207–353), which showed no auto-activation (see Materials and Methods), was used as a bait (Fig. 2). All grown colonies that showed a strong blue color in the subsequent β-galactosidase filter assay had their plasmid DNA extracted and sequenced. A total of 166 plasmid DNAs (69 from human bone marrow and 97 from human fetal brain cDNA libraries) from clones positive for both *HIS3* and *LacZ* reporters were sequenced. 59 different proteins were identified using FEZ2 (207–353) as a bait (Table 1, Fig. 3). These can be organized into the following groups according the major described GO (Gene-Ontology) process attributed to them: transcription (10 proteins), translation (6 proteins), apoptosis (5 proteins), signal transduction (5 proteins), neuronal cell development (5 proteins), cytoskeleton/centrosome (3 proteins), unknown function (10 proteins) and other functions (15).

**Table 1:** Human FEZ2-interacting proteins identified by the yeast two-hybrid system screen.

Gene	Acession no.	Protein Description^1^	Coded protein residues (complete)	Coded protein residues (retrieved)^2^	UNC76^3^	FEZ1^3^	FEZ2^3^	Biological Process (GO)^4^
AATF	NP_036270	apoptosis antagonizing transcription factor	560	11–319	+	+	+	apoptosis
ATP6V1H	NP_057078	H(+)-transporting two-sector ATPase	247	63–234	+	+	+	ion transport
C1orf216	NP_689587	hypothetical protein LOC127703	229	20–229	+	+	+	UNKNOWN
C10orf54	Q9H7M9	Platelet receptor Gi24 precursor	311	215–311			+	UNKNOWN
C10orf78	NP_001002759	hypothetical protein LOC119392 isoform a	245	6–245	+	+	+	UNKNOWN
C12orf51	NP_001103132	AF-1 specific protein phosphatase	3996	2878–3161		+	+	protein modification
C14orf94	CAD62584	HAUS augmin-like complex, subunit 4	387	16–333	+	+	+	centrosome organization
CAP1	EAX07240	CAP, adenylate cyclase-associated protein 1 (yeast)	468	163–468	+	+	+	cytoskeleton/cell polarity/signal transduction
CDKN1	EAW96276	cyclin-dependent kinase inhibitor 1B (p27, Kip1)	53	1–53	+	+	+	apoptosis/cell growth
CHERP	BAD92967	calcium homeostasis endoplasmic reticulum protein	399	301–399		+	+	nervous development
DEFA3	AAA35753	neutrophil peptide 3 precursor	65	50–65	+	+	+	xenobiotic metabolism
DNAJB11	NP_057390	DnaJ (Hsp40) homolog, subfamily B, member 11 precursor	358	277–358			+	protein folding
DNML1	EAW88521	dynamin 1-like, isoform CRA_c	789	549–789	+	+	+	endocytosis
DRAP1	NP_006433	DR1-associated protein 1	205	1–133	+	+	+	transcription
DRG2	BAD92577	developmentally regulated GTP binding protein 2 variant	259	51–259	+	+	+	signal transduction
EEF1G	AAH21974	H sapiens eukaryotic translation elongation factor 1 gamma	355	19–346			+	translation
EI2B5	EAW78300	eukaryotic translation initiation factor 2B, subunit 5 epsilon	442	327–434	+	+	+	translation/hormone mediated signaling
FEZ1	EAW67636	fasciculation and elongation protein zeta 1 (zygin I)	392	131–371	+	+	+	nervous development
FEZ1	NP_072043	zygin 1 isoform 2	104	1–89			+	nervous development
GNAS	AAH89157	GNAS complex locus	380	293–380			+	signal transduction
GOLPH3L	EAW53527	golgi phosphoprotein 3-like	299	159–299	+	+	+	UNKNOWN
GPRASP2	NP_612446	G protein-coupled receptor associated sorting protein 2	838	496–812	+	+	+	UNKNOWN
GTF2IRD2	AAQ19673	general transcription factor II i repeat domain 2	949	565–603			+	transcription
GTPB6	NP_036359	pseudoautosomal GTP-binding protein-like protein	403	98–347			+	UNKNOWN
HSP90B1	EAW97723	heat shock protein 90 kDa beta (Grp94), member 1	367	1–287			+	apoptosis/protein folding/muscular contraction
HSPA1A	BAD93055	heat shock 70 kDa protein 1A variant	709	75–163			+	response to stress
IGL@	AAH71804	IGL@ protein	236	1–236			+	UNKNOWN
IK	EAW62028	IK cytokine, down-regulator of HLA II	557	119–333	+	+	+	cell-cell signaling
INPP1	NP_002185	inositol polyphosphate-1-phosphatase	399	355–399		+	+	signal transduction
INTS8	NP_060334	integrator complex subunit 8	995	508–816	+	+	+	snRNA proccessing
MCM7	NP_877577	minichromosome maintenance complex component 7	543	410–543	+	+	+	transcription/cell cycle
MED7	NP_004261	mediator complex subunit 7	233	116–233	+	+	+	transcription
MLF1IP	NP_078905	MLF1 interacting protein	418	243–263	+	+	+	transcription
NFS1	BAD96959	NFS1 nitrogen fixation 1 isoform a precursor variant	457	417–457			+	metabolic proccess
NQO2	CAI23293	NAD(P)H dehydrogenase, quinone 2	172	1–115			+	oxidation reduction
OS9	NP_006803	amplified in osteosarcoma isoform 1 precursor	667	389–635		+	+	endoplasmatic reticum stress
PDCD7	AAI31705	PDCD7 protein	270	57–270	+	+	+	apoptosis
PTN	EAW83871	Pleiotrophin	246	105–223	+	+	+	nervous development
RARA	EAW60657	retinoic acid receptor, alpha, isoform CRA_e	520	111–327	+	+	+	transcription
RPL27	EAW60910	ribosomal protein L27, isoform CRA_b	80	53–80	+	+	+	translation
RPL38	NP_000990	RPL38 ribosomal protein L38	70	1–70			+	translation
RPS25	NP_001019	ribosomal protein S25	125	1–114			+	translation
SAP30	NP_003855	Sin3A-associated protein, 30 kDa	220	1–220	+	+	+	transcription
SAP30L	NP_078908	SAP30-like	183	1–183	+	+	+	transcription
SCOC	EAX05102	short coiled-coil protein, isoform CRA_a	122	42–122	+	+	+	UNKNOWN
SERPINF1	P36955	Pigment epithelium-derived factor precursor (PEDF)	418	16–267			+	nervous development
SFRS8	EAW98526	splicing factor, arginine/serine-rich 8	951	263–511	+	+	+	transcription
SHMT1	BAD97272	serine hydroxymethyltransferase 1 (soluble) isoform 1 variant	483	364–483			+	hormone mediated signaling
SLC25A37	AAF71063	PRO1584	81	1–58	+	+	+	muscular constraction
SLTM	EAW77552	SAFB-like, transcription modulator	1168	389–723		+	+	transcription/apoptosis
SMC3	BAF98736	structural maintenance of chromosomes 3	1217	766–1059	+	+	+	sister chromatides cohesion/signal transduction
STRA13	NP_659435	stimulated by retinoic acid 13	63	3–63	+	+	+	UNKNOWN
TBC1D25	NP_002527	TBC1 domain family, member 25	688	498–677	+	+	+	regulation Rab GTPase activity
TLOC1	Q99442	Translocation protein SEC62	399	2–95			+	cotranslational protein targeting to membrane
TNNC2	NP_003270	fast skeletal muscle troponin C	160	1–137			+	muscular constraction
TSNAX	NP_005990	translin-associated factor X	290	1–276	+	+	+	cell differentiation
TUBA1B	BAF82043	tubulin, alpha 1b	451	285–432			+	cytoskeleton
WWC1	EAW61508	WW, C2 and coiled-coil domain containing 1	1018	775–1018	+	+	+	UNKNOWN
“SIH”	A61065	sucrase-isomaltase homolog – human	48	1–44	+	+	+	UNKNOWN

1 Results obtained from BLASTX (GenBank);

2 It is depicted the mininum length of the retrieved sequences which could be visualized by forward DNA sequencing only,

3 Interaction confirmed (+) by yeast two hybrid system with UNC-76, FEZ1 or FEZ2 proteins;

4 Biological process based on the GO database (other functions may be known).

Most interestingly, of these 59 FEZ2 interactors only 40 interacted also with FEZ1 (Fig. 3, Table 1) and indeed 8 of the 40 were identical with proteins that had been identified in the screen with FEZ1 [10]. This result may suggest that FEZ2 gained in respect to FEZ1 additional 19 new interactors, within the set of preys analyzed, which are specific to FEZ2. Such an evolutionary gain of function is compatible with the histological expression patterns of both proteins. Whereas FEZ1 (as its ortholog UNC-76) is expressed only in the nervous system, FEZ2 shows a ubiquitous expression pattern. Thinking of the role of FEZ proteins as transport adaptor proteins, FEZ2 may have evolved to transport additional cargo proteins, which may be expressed in non-neuronal tissues.

### UNC-76 and FEZ1 share a conserved protein-protein interaction pattern

Next we were interested to involve UNC-76 as a third component in our analysis, to try to understand how the protein-protein interaction pattern may have evolved during evolution of the FEZ family proteins. To this end we employed a C-terminal construct of UNC-76 (242–378) in a direct one-on-one analysis against all 59 FEZ2 interacting proteins (Fig. 3, Table 1). The cDNA for *C. elegans* UNC-76 was a kind gift from Dr. Hengartner.

To our great surprise, the pattern of interaction of UNC-76, in relation to the proteins identified in the screening assay of two-hybrid and FEZ2 as bait, was almost identical to that observed for FEZ1 (Table 1, Fig. 3). This suggests that the protein-protein interaction profile of the orthologs FEZ1 and UNC-76 is highly conserved. Only 4 of the 40 proteins that interacted with FEZ2 and FEZ1 did not interact with UNC-76. In conclusion the protein interaction pattern for both human FEZ1 and *C. elegans* UNC-76 seemed to be mostly conserved since the divergence of the deuterostomes (represented by the human FEZ1) from the “protostomes” (represented by *C. elegans* UNC-76), which occurred around 650 million years ago.

**Figure 4:**
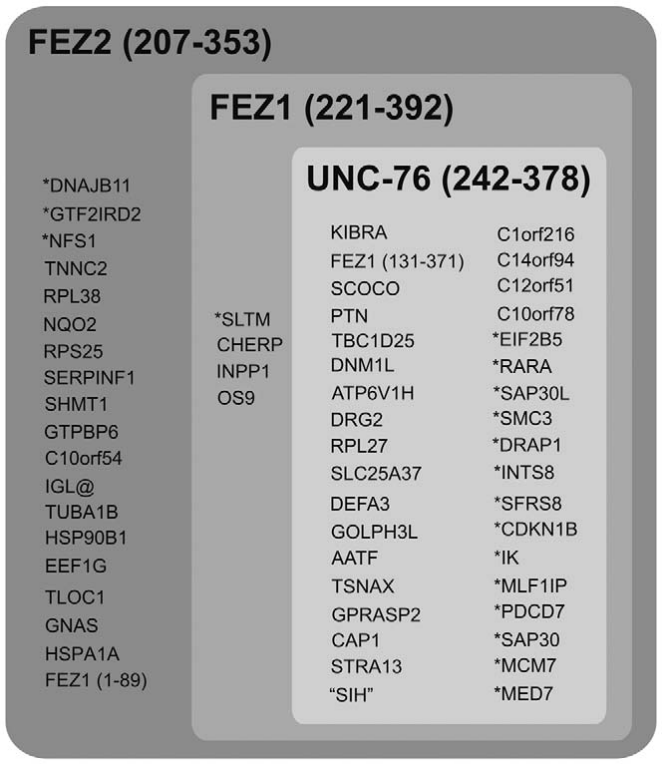
Schematic summary of the individual interactors of FEZ1, FEZ2 and UNC-76. On top of the boxes, highlighted by larger print and in bold, are the bait proteins used in the yeast two-hybrid assays. Sets are represented by gray boxes. The darker the gray the larger the set of proteins. Nuclear proteins are indicated by an asterisk preceeding the official protein name.

In this context it is worthwhile to differentiate between nuclear and cytoplasmic proteins that interact with the FEZ family proteins. A significant proportion of the interacting proteins are nuclear or predicted to be nuclear (18 of 59, 30.51%). Among these 18 nuclear proteins identified in the screen with FEZ2, a high proportion of 15 (ca. 83%) also interacted with FEZ1 (Fig. 4). This may be of potential relevance because we have shown in previous cell fractionation experiments that GFP-FEZ1 can be found in the nuclear fraction [26]. The exact role of FEZ1 in the nucleus remains however to be established. Interestingly, of the 41 cytoplasmic FEZ2 interacting proteins identified, a significant fraction of 16 (ca. 40%) interact exclusively with FEZ2. This again may suggest the acquisition of new interaction partners and thereby possibly the aggregation of new “cytoplasmic functions” for FEZ2 relative to FEZ1/UNC-76.

In 2000, Walhout and collaborators [28] introduced the concept of an “interologue”, referring to a protein-protein interaction pattern that is conserved between pairs of orthologs. This notion is intuitive in molecular biology and has been documented by many interactions and may be conserved even in distantly related organisms, such as bacteria and animals. Our results show not only a high degree of preservation of the interactions between FEZ1, FEZ2 and UNC-76 but also the acquisition of new PPIs for FEZ2. Makino and coworkers [29] suggested that the rate of evolution of a protein that has more partners PPI is much smaller than the one that has few partners PPI. This is in good agreement with the high degree of sequence similarity among FEZ1/FEZ2/UNC-76, which is especially pronounced in the C-terminal coiled-coil region, which is essential for most protein-protein interactions (Figure S2). Only 12.8% of the amino acids in the coiled-coil regions of FEZ1 and FEZ2 are different.

Three different fates have been proposed after the duplication of a genes [29]. First, one of the copies can be silenced by the accumulation of deleterious mutations and eventually become indistinguishable from genomic non-coding neighboring regions, while the other copy retains the original function. Second, while one copy maintains the original function, the other acquires a new function possibly by aggregation of advantageous mutations. Third, both copies accumulate mutations that alter the original function, but the original function is retained and compensated cooperatively. Our results seem to strongly suggest that for the FEZ family the second pathway is in progression. All interactors identified for FEZ1 in our laboratory have been confirmed to interact with FEZ2, too [10], but only 60% of the interactions identified for FEZ2 were confirmed for FEZ1 and UNC-76. Thus, FEZ2 seems to have acquired additional new functions but still preserved the majority of interactions found for FEZ1/UNC-76.

In this context it is also interesting to analyze the origin of the proteins fished with the FEZ2 bait from either bone marrow or fetal brain cDNA libraries and to correlate this with the supposed expression patterns (FEZ1: brain, FEZ2: ubiquitous) and function (FEZ1: neuron specific, FEZ2: ubiquitous) (Table 1). Of the 17 proteins that were of origin from either bone marrow or brain (not from both, like 2 additonal proteins) and that were specific to interact with FEZ2, 13 were from bone marrow and only 4 from brain. Of the 52 proteins that were of origin from either bone marrow or brain only (7 additional proteins were fished from both libraries!) and that interacted with both FEZ1 and FEZ2, 32 were from the bone marrow library and 20 from the brain. Thus, FEZ1 interactions are relatively enriched in clones from the brain, while FEZ2 interactions are of more ubiquitous origin. In fact the ratio bone marrow proteins/brain protein for FEZ2 is almost three times higher (3.25; 13/4) than for FEZ1 (1.19; 19/16). This tendency may already indicate the acquisition of additional interacting partners and functions in the more ubiquitously expressed FEZ2 relative to the brain specific FEZ1 since the candidate cargo proteins for transport of FEZ1 can be mostly found in brain tissue, whereas those of FEZ2 preferentially in other tissues, including bone marrow.

## Discussion

The large number of proteins that were identified to interact FEZ1, led to its classification as a hub protein [10]. Most proteins initially characterized as hubs tend to maintain this status after additional studies [30]. Indeed, here we were able to confirm the hub status for human FEZ1 and even extend it to the entire family of FEZ proteins, since both human FEZ2 as well as *C. elegans* UNC-76 interacted also with a large number of proteins. Genome-wide studies have shown that deletions of hub protein encoding genes are three times more likely to be lethal than deletions of non-hubs, a phenomenon known as the centrality-lethality rule [31]. This is confirmed in *C. elegans* mutations in the *unc-76* gene, which although not lethal, cause deleterious abnormalities in the elongation of neuronal axons along other axonal surfaces (but not over non-neuronal surfaces) during the animals development, which results in a “paralyzed” phenotype, where the worm has severly reduced body movements (therefore the designation unc for uncoordinated movements). These abnormalities can be complemented by the expression of human FEZ1 [1]. This result already shows the high functional conservation which we confirmed and detailed here: the protein interaction profiles of FEZ1 and UNC-76 are highly overlapping, since 36 of 40 FEZ1 interactors also interact with UNC-76 (90%).

In this context it is also interesting to note that although the knockout of UNC-76 had severe and deleterious effects in the worm a recently described knockout mice for FEZ1 showed no dramatic changes in the brains anatomy or, the morphology of neuronal cell bodies, dendrites or axons, nor of the general locomotion or behavior of the animals [32]. Only after new social interactions or under stress conditions a slight hyper locomotion behavior was observed as well as a greater response to certain psycho-stimulants [32]. Our comparative analysis of the FEZ1 and FEZ2 interactomes becomes interesting at this point, because in mammals, and chordates in general, we have two FEZ family gene members in contrast to the *C. elegans* genome, which contains only *unc-76*. The duplication of the *unc-76* gene occurred most likely in the stemline of the chordates (Table S1, Fig. 1) [6]. Our current interpretation of the lack of any greater neurophysiological or behavioural abnormalities in the *FEZ1*-/- mice, may be due to a compensatory expression of the ubiquitously expressed FEZ2, which - as we demonstrated here - not only acquired additional new interactors in respect to FEZ1, but at the same time also maintained **all** of the interactors of FEZ1. From our point of view it is therefore not at all surprising that the FEZ1-/- mice had such a mild phenotype in comparison to the UNC-76 -/- worm [32]. It may even be that expression of FEZ2 in the FEZ1 k.o. mice, by hitherto unknown mechanisms, may be up-regulated in comparison to wild-type mice. It is interesting to note that in Figure S1A of the article of Sakae and co-workers, we can observe a new band at the height of the FEZ1 protein in extracts from the stomach of FEZ1-/- but not wild type mice (anti-FEZ1 Western blot). Since FEZ2 has almost the same MW as FEZ1, this band may represent up-regulated FEZ2 protein, in response to the lack of FEZ1. The observed relatively mild phenotype in the *FEZ* -/- mice may be as well attributed to additional proteins interacting with FEZ2, which may interfere in the way of a dominant negative in FEZ1 specific functions, as to specific but not yet identified PPI for FEZ1.

Although FEZ2 has more interactors than FEZ1 there is not a 100% functional redundancy. This would explain why both genes are still maintained in the gene pool (from a population genetics point of view). The reasons why both are maintained may be manifold, including dosage effects or a promoter specific regulation that must be maintained in the neurons (FEZ1). Data from the literature suggest that FEZ1 expression is increased upon NGF treatment, a neuronal tissue specific factor [6], [33]. It may be even speculated that FEZ1 is somehow involved in neurogenesis per se. FEZ1 mRNA expression shows a peak that coincides with neurogenesis in rats, and decreases with the progress of adulthood. The mRNA expression FEZ2, although with higher levels in embryogenesis, is relatively constant, always less than that of FEZ1. Additional data on FEZ2 however are not available so far but one could speculate that its more ubiquitous expression depends on internal genetic programs rather than on external stimuli, like in the case of FEZ1.

Many of the proteins identified by our screen with FEZ2 interact with proteins that also interact with each other, increasing therefore the likelihood that these are true interactions of biological relevance. We like to highlight here the interactions of pleiotrophin (PTN), protein tyrosine phosphatase, receptor type S (PTPRS) ([18], and retinoic acid receptor alpha (RARA). In 2005, Stelzl et al. [18] performed a yeast two-hybrid screen on a large scale, reporting more than 3000 interactions between human proteins. The human protein FEZ1 interacted with 21 proteins. Of these 21 proteins, 4 interact with pleiotrophin (general transcription factor IIF, protocollagen hydroxylase, neurokinin beta, translocase of outer mitochondrial membrane 20 homolog). FEZ1 interacted further with a receptor protein tyrosine phosphatase (PTP) (sigma) of the same family of receptors of pleiotrophin (beta and zeta) [34]. Pleiotrophin is also known as neurite growth-promoting factor 1 (NEGF1), has mitotic activity and influences the growth of neuritis. It acts in the developing nervous system [35] and signals via the inactivation of a receptor-dependent ligand of RPTP beta/zeta (or PTPRS). It increases the level of tyrosine phosphorylated signaling proteins in the cell, promoting thereby downstream signaling events [34]. In the nervous system, PTN is expressed in both neurons and glia cells [36] as well as in neuronal stem cells [37]. SAGE analysis indicated a 10-fold elevation of PTN transcripts in response to long-term NGF treatment of PC12 cells. It is of interest, that in P19 neuroprogenitor cells, PTN was also induced by retinoic acid during neuronal differentiation [38], [39]. Retinoic acid is essential for the differentiation and morphogenesis of various tissues, including the nervous system [40]. Silencing of FEZ1 by RNA interference (RNAi) strongly inhibited the NGF-induced differentiation and efficiently reduced the anterograde transport of mitochondria in PC12 cells, suggesting that FEZ1 is essential for NGF-induced neuronal differentiation of PC12 cells [33], [41].

Retinoic acid receptors (RARs) are transcriptional activators which are activated by binding of their ligands and act in concert with a combination retinoid X type receptors (RXR alpha, beta and gamma). Even in the absence of ligand, RAR-RXR heterodimers bind to DNA sequences known as response elements for RA, but in this state they recruit co-repressors. These co-repressors mediate the negative modulation of transcription by recruitment of the histone deacetylase complex and the transfer of methyl radicals to the DNA bound histones, thereby stabilizing the nucleosome. The binding of the ligand retinoic acid causes however conformational changes in the binding domain of RAR, which in return promote the release of the co-repressors and the recruitment of co-activators. While some co-activators interact with factors of the basal transcription machinery, others induce chromatin remodeling and specific transcriptional activation. Data from our previous two-hybrid and pull-down assays confirmed the interaction of FEZ1 and FEZ2 proteins related to activation of transcription (BAF60a) and the repression of transcription (SAP30L) [10]. Furthermore, RXR/RAR interacts with N-Cor, thereby recruiting the complex of repression of transcription [42]. It is known that SAP30 also interacts with N-Cor [43] promoting the compaction of chromatin. These data gain potential relevance by the observation that both RAR and FEZ1/FEZ2 interact with protein complexes of chromatin remodeling, and with each other. Thus, FEZ1 may be also involved in the regulation of gene transcription mediated by transcription factors.

In summary, we have described here the molecular expansion of the FEZ protein family in the chordate stemline, most likely after two rounds of whole-genome duplication. We have identified many new members of the FEZ family from different species and a highly conserved C-terminal domain, which is responsible to mediate the majority of the protein-protein interactions. Our analyses indicate that the ancestral UNC-76 protein function and protein interaction profile is surprisingly conserved in animals, evolutionary distinct as vertebrates and nematodes. The analysis of the phylogeny and the detailed protein interaction profiles uncovered a likely functional divergence between FEZ1 and FEZ2, since FEZ2 acquired in respect to FEZ1/UNC-76 additional new interactors and is ubiquitously expressed and not restricted to neuronal cells, as is FEZ1/UNC-76. Our data provide an explanation for the ability of human FEZ1 to rescue the defects caused by *unc-76* mutations in nematodes, since the PPI pattern of FEZ1 and UNC-76 are highly similar. Furthermore, no strong defects are caused in mice lacking the *FEZ1*, probably due to the compensatory presence of its paralogue FEZ2, which interacted with all FEZ1 interacting proteins, and additional new interactors that are FEZ2 specific. Our new data will hopefully stimulate and facilitate further studies on the functional role of proteins of the FEZ family in the development and function of both neuronal and non-neuronal cells. Furthermore, studies such as ours may contribute to our ever growing understanding of how protein networks function and specially how they came about and were modified and adapted during evolution. In the future we may devise new and more complete ways to visualize and comprehend how these networks were shaped throughout time.

## Supporting Information

Figure S1 **Amino acid sequence alignment of the members of the FEZ family.** Names of the sequences are given in the Table S1. The residues in the alignment are shaded light grey, grey, or black to indicate shared identity in 40%, 70% or 100% of the analyzed sequences, respectively. The bars indicate regions predicted to form coiled-coil.
(PDF)

Figure S2 **Identities and similarities between human proteins FEZ1 and FEZ2 and** ***C. elegans*** **UNC-76.** A general scheme of the FEZ family proteins is shown at the top. The identity and similarity comparisons were made of two-by-two proteins both for the complete protein alignment as well as for local alignment of FEZ fragments by NPS@ (http://npsa-pbil.ibcp.fr/cgi-bin/npsa_automat.pl?page=npsa_clustalw.html). I = identity, S = strongly similar, W = weakly similar, D = different.
(TIF)

Table S1 **FEZ family proteins used for amino acid sequence alignment.**
(DOC)
