# Supplementary material for: FEZ2 Has Acquired Additional Protein Interaction Partners Relative to FEZ1: Functional and Evolutionary Implications
Source: PLoS One. 2011 Mar 8;6(3):e17426. doi: 10.1371/journal.pone.0017426 (PMC3050892; doi:10.1371/journal.pone.0017426)
Supplement: Figure S1 — Amino acid sequence alignment of the members of the FEZ family. Names of the sequences are given in the Table S1. The residues in the alignment are shaded light grey, grey, or black to indicate shared identity in 40%, 70% or 100% of the analyzed sequences, respectively. The bars indicate regions predicted to form coiled-coil. (PDF) [file pone.0017426.s001.pdf]

```
                      *        20         *        40         *        60         *        80         *       100
Pt_FEZ1     : --------------------------------------------------------------MEAPLVSLDEEFEDLRPSCSEDPE------------------- : 24
Hs_FEZ1     : --------------------------------------------------------------MEAPLVSLDEEFEDLRPSCSEDPE------------------- : 24
Mf_FEZ1     : --------------------------------------------------------------MEAPLVSLDEEFEDLRPCCSEDPE------------------- : 24
Mmt_FEZ1    : --------------------------------------------------------------MEAPLVSLDEEFEDLRPCCSEDPE------------------- : 24
Bt_FEZ1     : --------------------------------------------------------------MEAPLVSLDEEFEDLRPCCSEDPE------------------- : 24
Cf_FEZ1     : --------------------------------------------------------------MEAPLVSLDEEFEDLRPCCSEGQE------------------- : 24
Rn_FEZ1     : --------------------------------------------------------------MEAPLVSLDEEFEDIRPCCTEDPE------------------- : 24
Mm_FEZ1     : --------------------------------------------------------------MEAPLVSLDEEFEDIRPSCTEEPE------------------- : 24
Md_FEZ1     : --------------------------------------------------------------MEAPLVSLDEEFEDLRPCYSEDRD------------------- : 24
Xt_FEZ1     : --------------------------------------------------------------MEAPLVCLDEEFEDIR-SYSEDRQ------------------- : 23
Dr_FEZ1     : --------------------------------------------------------------MEAPLVCLDEEFEDLRPCKMEELEC------------------ : 25
Ss_FEZ1     : --------------------------------------------------------------MEAPLVCLDEEFEDLRPCRVEDMDR------------------ : 25
Gg_FEZ1     : --------------------------------------------------------------MEAPLVSLEEEFEEGP--------------------------- : 16
Xt_FEZ2     : -------------------------------------MAAPLAQFDEDWQDFYEFRAASSSDSGCLDKVNSNTPSAVAPLL------------------------ : 44
Xl_FEZ2     : -----------------------------------------------------------------LDKVNSNPPPSESRIQ------------------------ : 16
Cf_FEZ2     : -------------------------------------------------------------------MAADGDWQDFYEFQ------------------------ : 14
Bt_FEZ2     : -------------------------------------------------------------------MAADGDWQDFYEFQ------------------------ : 14
Hs_FEZ2     : -------------------------------------------------------------------MAADGDWQDFYEFQ------------------------ : 14
Pt_FEZ2     : -------------------------------------------------------------------MAADGDWQDFYEFQ------------------------ : 14
Rn_FEZ2     : -------------------------------------------------------------------MAADGDWQDFYEFQ------------------------ : 14
Mm_FEZ2     : ---------------------------------------------------------------AAGAMAADGDWQDFYEFQ------------------------ : 18
Md_FEZ2     : -------------------------------------------------------------------MAADGDWQDFYEFQ------------------------ : 14
Tg_FEZ2     : MAAQEKKYKIKKSHPKLLRVSAQYKASKGWARESQCWSLRTQTTLGLPLLPDEPPLVGAELGVRADTAVPGSKGAATARLCGCCPSPARPGPALRA--------- : 96
Ec_FEZ2     : ----------------------------------------------------------------MDNSSELTQWHTMWDWC------------------------ : 17
Tn_FEZ2     : --------------------------------------------------------------MAAPLAQFDEDWQDFNEFKP----------------------- : 20
Dr_FEZ2     : --------------------------------------------------------------MAAPVAQFDEDWEDFNEFK------------------------ : 19
Bf_FEZ2b    : --------------------------------------------------------MMESFKMAAPLAQIDDEWLDFTSPS------------------------ : 25
Bf_FEZ2a    : --------------------------------------------------------------MAAPLAQIDDEWLDFTSPS------------------------ : 19
Ci_FEZ1sim  : --------------------------------------------------------------MAAPLAQIEDEWESFASFSPTRLN------------------- : 24
Ce_UNC76    : ------------------------------------------------------MEAADLRVPDIPLASCDDD-------------------------------- : 19
Bm_UNC76    : -------------------------------------------------MGTGMVENINCSVPEVPLAHLEDD-------------------------------- : 24
DPs_UNC76   : ---------------------------------------------------------MAELKFEAPLAKFEET-DEWGGCDFISNQNALNDTLNLN---LKEVSV : 44
Dp_UNC76    : --------------------------------------------------MRDLGTKMAELKFEAPLAKFEET-DEWGGCDFISNQNALNDTLNLN---LKEVSV : 51
Ds_UNC76    : ---------------------------------------------------------MAELKFEAPLAKFEET-DEWGGCDFISSQNAINDTLNLN---LKDSSA : 44
Dm_UNC76    : --------------------------------------------------MRDLGTKMAELKFEAPLAKFEET-DEWGGCDFISSQNAINDTLNLN---LKDSSA : 51
Dy_UNC76    : --------------------------------------------------MRDLGTKMAELKFEAPLAKFEET-DEWGGCDFISSQNALNDTLNLN---LKDSSS : 51
Da_UNC76    : --------------------------------------------------MRDLGTKMAELKFEAPLAKFEET-DEWGGCDYISNQNALNDTLNLN---LKDAAA : 51
Dw_UNC76    : --------------------------------------------------MRDLGTKMAELKFEAPLAKFEET-DDWGGCDFISNQNALNDTLNLN---LKD--- : 48
Dv_UNC76    : ---------------------------------------------------------MAELKFEAPLAKFEET-DEWGGCDFISNQNALNDTLNLNLSSNRNQKD : 47
Dmo_UNC76   : --------------------------------------------------MRDLGTKMAELKFEAPLAKFEET-DEWGGCDFISNQNALNDTLNLNLSSNRNQKE : 54
Dg_UNC76    : ---------------------------------------------------------MAELKFEAPLAKFEET-DEWGGCDFISNQNALN--------ANRNLKD : 39
Tc_UNC76    : --------------------------------------------------MRDLATKMAELKFEAPLAQFEES-DEWGSTEFQNATIKN---------------- : 38
Ag_UNC76    : --------------------------------------------------MRDLANKMAELKFEAPLAQFEES-DEWGPVEYQSSNVANGKTAAVT--------- : 45
Nv_UNC76    : --------------------------------------------------MKDMATKIAELKFEAPLACFEEE-E----STTIRN-------------------- : 30
Am_UNC76    : --------------------------------------------------MRDMAGKIAELKFEAPLARFEEE-D----TASLKN-------------------- : 30
Ph_UNC76    : --------------------------------------------------MLDTARQMAELKFEAPLAQFEESEDEWAIGGMTINTNDVVDN------------- : 42
Ap_UNC76    : --------------------------------------------------MLDMMNKMAELKFEAPLAQFEES-DEWTK--MMDDDSPLQQS------------- : 39
```

```
                    *        120      *        140      *        160      *        180      *        200      *
Pt_FEZ1     : ----------EKPQCFYGSSPHHLEDPS------------------------------------------------------------------LSELENFSS-- :  51
Hs_FEZ1     : ----------EKPQCFYGSSPHHLEDPS------------------------------------------------------------------LSELENFSS-- :  51
Mf_FEZ1     : ----------EKPRCFYGSSPHHLEDPS------------------------------------------------------------------LSELENFSS-- :  51
Mmt_FEZ1    : ----------EKPRCFYGSSPHHLEDPS------------------------------------------------------------------LSELENFSS-- :  51
Bt_FEZ1     : ----------EKPRCFYGSSPHHLEDPS------------------------------------------------------------------LSELENFSS-- :  51
Cf_FEZ1     : ----------EKPPSFYGSSPHHLEDPS------------------------------------------------------------------LSELENFSS-- :  51
Rn_FEZ1     : ----------EKPQSLYGTSPHHLEDPS------------------------------------------------------------------LSELENFSS-- :  51
Mm_FEZ1     : ----------EKPQCLYGTSPHHLEDPS------------------------------------------------------------------LSELENFSS-- :  51
Md_FEZ1     : ----------EKPRCFYGSSPHHLEDPS------------------------------------------------------------------LSELENFSS-- :  51
Xt_FEZ1     : ----------EKSRGMYMTSSKHIEDAS------------------------------------------------------------------LSELENFSS-- :  50
Dr_FEZ1     : ----------EQPPCR-PHKTIPLAPLC------R---------------------------------------------------------EDFSELENFS--- :  53
Ss_FEZ1     : ----------AVPLSRPPYSTIPLAPMAPLAPITR---------------------------------------------------------EDFSELENFS--- :  60
Gg_FEZ1     : --------------GDNGGVPRRTMDPA------------------------------------------------------------------LAELESFST-- :  39
Xt_FEZ2     : --------GQG-AGAVVALEDLAELDN-------------------------------------------------------------------GFSMEIMG--- :  70
Xl_FEZ2     : --------GPGGAGAVVALEDVAELDN-------------------------------------------------------------------GFSMEIMG--- :  43
Cf_FEZ2     : ----------EPARSLQDQENCNASP--------------------------------------------------------------------EAGAGPGG--- :  38
Bt_FEZ2     : ----------EPARSLQDQENCNASP--------------------------------------------------------------------EAGAGPGG--- :  38
Hs_FEZ2     : ----------EPARSLLDQENCNASPEP----------------------------------------------------------------GAEAGAGAGG--- :  42
Pt_FEZ2     : ----------EPARSLLDQENCNGSPEP----------------------------------------------------------------GAGAGAGAGW--- :  42
Rn_FEZ2     : ----------EPAGSVRDQENCNASP--------------------------------------------------------------------EAGAGAHA--- :  38
Mm_FEZ2     : ----------EPAGSVQEQENCNASP--------------------------------------------------------------------EAGAGAHA--- :  42
Md_FEZ2     : ----------EPARGRLDQENRNSGA----------------------------------------------------------------------KMLDDE--- :  36
Tg_FEZ2     : --------IARPGPARLCGRCLSPARRAPLGARTAGSA------------------------------------------------------QHRERAAPRSQLW : 139
Ec_FEZ2     : -----------PAEAEVLRRNLGSQKKQKLKELDS----------------------------------------------------------KHSKASPWS--- :  50
Tn_FEZ2     : ---------SSASADQLDQLNSNVVD-------------------------------------------------------------------SASGLDDFSD-- :  47
Dr_FEZ2     : ------------AADSHTSWTATPEN-------------------------------------------------------------------SPR-LQSFAS-- :  42
Bf_FEZ2b    : ----------DVVNSNDTTSSCTDDNQD------------------------------------------------------------------VDNVDNCGFG- :  53
Bf_FEZ2a    : ----------DVVNSNDTTSSCTDDNQD------------------------------------------------------------------VDNVDNCGFG- :  47
Ci_FEZ1sim  : ----------MCSTNVEQLEDVKNQNMSFGNS------------------------------------------------------------ENLDSIDNFIPD- :  58
Ce_UNC76    : ----------DIDSNKN--------------------------------LSNHSSDEKH------------------------------------HCNSNSDEE- :  45
Bm_UNC76    : -----------LDFIKV--------------------------------ASQQSTSD---------------------------------------CENNFDEN- :  46
DPs_UNC76   : GRKP---DASKLRLLEDAVRDAHISKNGGV----------------GSSAG-TISPNCNSLLSGSATDLGVSDAGLVPGDGA-----GLDKPSAAQG-DHVDN-- : 121
Dp_UNC76    : GRKP---DASKLRLLEDAVRDAHISKNGGV----------------GSSAG-TISPNCNSLLSGSVTDLGVSDAGLVPGDGE-----VLDKPSAAQG-DHVDN-- : 128
Ds_UNC76    : GGKP---DSTKLRLLEDAVRDAHVSKNGGG--------------VVGGAAG-SISPNCNTLHGGSLIEIGLSDVGLVPGEGAGVGLDGLEKRSLAVG-DHVDN-- : 128
Dm_UNC76    : GGKP---DATKLRLLEDAVRDAHVSKNGGG--------------VVGGGAG-SISPNCNTLQGGSLIEIGLSDVGLVPGEGAGVGLDGLEKRSLAVG-DHVDN-- : 135
Dy_UNC76    : GGKP---DAAKLRLLEDAVRDAHVSKNGGGGG------------VMGGGAG-SISPNCNTLQGGSLIEIGLSDVGLVPGEGAGVGLDGLEKRSLAAG-DHVDN-- : 137
Da_UNC76    : SGKSGQDTAGKLRLLEDAVRDAHVSKNGGVDAA-----------AAGASIGGSISPNCNTMQG--ALDLGLSDVGLVPGELA-------PQRCGGSG-DHVDN-- : 133
Dw_UNC76    : GGKVETAANNKLRLLEDAVRDAHVSKNGGAG--------------AGTGSG-SISPNCNSMGGG--TDLGISDVGLVPGDGAG----GAAAGSARSGSDHVDN-- : 130
Dv_UNC76    : GNAKQQQQ-DKLRLLEDAVRDAHISKN--------GGGGGGG----GAGAG-NISPNCNTHNLG---ELGLADM---SGPAGG---------QDKRGADNVDN-- : 121
Dmo_UNC76   : GSAKQQQQQDKLRLLEDAVRDAHISKNGAVATGAVGGAGAGAGTGTGTGAG-NISPNCNTQNLS---ELGLTD-----GLAGG---------QDKRGADNVDN-- : 139
Dg_UNC76    : GNAKQQQQ-DKLRLLEDAVRDAHLSKNGGAAAAVSGAGAAGSG---SGTSG-NISPNCNTHNLSD--VLGLATPGVGLGIGLG---------GDRRAADNVDN-- : 126
Tc_UNC76    : ------ENELNNINLNKKIKDIN----------------------------DVLNDFNEDILN---------NSNTETLPPKN------VILKNGETASVADN-- :  92
Ag_UNC76    : -----ISDTLNLNNLKESLRSLDGNQQQQQ----------------QNQKDDDLNELNDNLLLGDDTVRGGGDSNTTTTTTTA------NNNNNIKVGPIKDNV- : 122
Nv_UNC76    : -----------MNIFSDHILDVNLERNFVE------------------------NRKTNTTAN-----------------------------THET-SDIIQE-- :  68
Am_UNC76    : -----------MNLLTEQLLDTSLNTTYGE------------------------NCNANEPAT-----------------------------TRENGTDILQE-- :  69
Ph_UNC76    : ----------FKNILNSSPLKKNINENFTN--------------------NIKNSKSGIFISNKGEINNMDFAYEKINGYSKE-----DMLKNLNQGNSVSEN-- : 110
Ap_UNC76    : ----------AKHNLHNHHHHHQGNSQLTSG---------------GSVRDVDEEQLDNMLSNSS--RALEPANKSCTQCGAN-----NNNVGVEVTTSDTDN-- : 110
```

```
                      220         *         240         *         260         *         280         *         300         *
Pt_FEZ1      : --------EIISFKSMEDLVNEFDEKLNVCFRNYNAKTEN------LAPVKNQLQIQEEEETLQ--------------DEEVWDALTDNYIP-SLSEDWRDPNI- : 126
Hs_FEZ1      : --------EIISFKSMEDLVNEFDEKLNVCFRNYNAKTEN------LAPVKNQLQIQEEEETLQ--------------DEEVWDALTDNYIP-SLSEDWRDPNI- : 126
Mf_FEZ1      : --------EIISFKSMEDLVNEFDEKLNVCFRNYNAKTEN------LAPVKNQLQIQEEEEALQ--------------DEEVWDALTDNYIP-SLSEDWRDPNI- : 126
Mmt_FEZ1     : --------EIISFKSMEDLVNEFDEKLNVCFRNYNAKTEN------LAPVKNQLQIQEEEETLQ--------------DEEVWDALTDNYIP-SLSEDWRDPNI- : 126
Bt_FEZ1      : --------EIISFKSMEDLVNEFDEKLNVCFRNYNAKTEN------LAPVKNQFQIQEEEETLQ--------------DEEVWDALTDNYIP-SLSEDWRDPNM- : 126
Cf_FEZ1      : --------EIISFKSMEDLVNEFDEKLNVCFRNYNAKTET------LAPVKNQFQIQEEEETLQ--------------DEEVWDALTDNYIP-SLSEDWRDPNM- : 126
Rn_FEZ1      : --------EIISFKSMEDLVNEFDEKLNVCFRNYNAKTEN------LAPVKNQLQIQEEEETLR--------------DEEVWDALTDNYIP-SLSEDWRDPNI- : 126
Mm_FEZ1      : --------EIISFKSMEDLVNEFDEKLNVCFRNYNAKTES------LAPVKIQLQIQEEEETLR--------------DEEVWDALTDNYIP-SLSEDWRDPNI- : 126
Md_FEZ1      : --------EIISFKSMEDLVNEFDEKLNVCFRNYNAKTEN------LAPVKNQLQIQEEEENLQ--------------DEEVWDALTDNYIP-SLGEDWRDPNI- : 126
Xt_FEZ1      : --------EIISFKSMEDLVNEFDEKLNVCFRNYNAKTEN------LAPVKNQLQIQEEEERLR--------------DEEVWDALTDNYIP-LTGETWRDQSL- : 125
Dr_FEZ1      : --------EMMSFKSMEDLVNEFDEKLNVCFHNYNTKTEG------LAPVRNQSHAEEDEERLQ--------------DEDVWDALTDNYMP-SSVSSWDDPTS- : 128
Ss_FEZ1      : --------EMMSFKSMEDLVNEFDEKLNVCFHNYNTKTEG------LAPVRNQSHTEEDEERLQ--------------DEDVWDALTDNYVP-SSVSSWDDPNS- : 135
Gg_FEZ1      : --------EMMSFKSMEDLVQEFDEKLTVCFRNYDATTEG------LAPVRGRLQAQEEEEHLQ--------------DEEVWDALTDGFVPRGSPRPWMHPEA- : 115
Xt_FEZ2      : -----------GFKSMEDLVNEFDEKLSVCFRNYST----------DTGTIAPVRPITEEGMMK--------------DDEIWNALTDNYGN-VMPVDWKTSHAR : 139
Xl_FEZ2      : -----------GFKSMEDLVNEFDEKLSVCFRNYST----------DTGTIAPVRPITEEGIMK--------------DDQMWNALTDNYGN-VMPVDWKTSHAR : 112
Cf_FEZ2      : -----------GGDGFPALACSLEEKLSLCFRPSGP---------GAEPPRAAVRPITECSLLQ--------------GDEIWNALTDNYGN-VMPVDWKSSHTR : 108
Bt_FEZ2      : -----------GGDGFPALACHLEEKLSLCFRPSGP---------AAEPPRAAVRPITECSLLQ--------------GDEIWNALTDNYGN-VMPVDWKSSHTR : 108
Hs_FEZ2      : -----------GADGFPALACSLEEKLSLCFRPSDP---------GAEPPRTAVRPITERSLLQ--------------GDEIWNALTDNYGN-VMPVDWKSSHTR : 112
Pt_FEZ2      : -----------GRRRFPGPVCSMSEQSLICIYSSSPL-------LALPPEPSPVRSATAATVLQ--------------TSEIWNALTDNYGN-VMPVDWKSSHTR : 114
Rn_FEZ2      : -----------GGDSFPALASSLEEKLSLCFRPTSD----------ADPPRAAVRPITERSLLQ--------------GDEIWNALTDNYGN-VMPVDWKSSHTR : 107
Mm_FEZ2      : -----------GGDSFPALASSLEEKLSLCFRPTSE----------AEPPRAAVRPITECSLLQ--------------GDEIWNALTDNYGN-VMPVDWKSSHTR : 111
Md_FEZ2      : -----------GGVGFSALACGLEEKLSLCFRPSSP---------DTESPRAAVRPITERSLLQ--------------EDEIWNALTDNYGN-VMPVDWKSSHTR : 106
Tg_FEZ2      : RRQLRPRSETRAIGRISRDSSPPRERPRACLRQGRLGRRGSRGEAVPTASPGPADPGGACGLRQRSLSPPPGLQPGAAGPRIWNALTDNYGN-VMPVDWKSSHTR : 243
Ec_FEZ2      : -------KGDRAVN-IREDSGTERDPNAARLRTG------------PRGPPRGESSRALCGR----------------GARIWNALTDNYGN-VMPVDWKSSHTR : 118
Tn_FEZ2      : -----------LDNSFSGEICSFKSMEDLVHDFDEKLTVCFRNYNTTTEDIAPVKPITEDNYLK--------------DDEVWNTLTDNYGN-VMAVDWKTSHTR : 126
Dr_FEZ2      : -----------LDETLS---ASFP-------------------YSCSACTNDSVTAVSESELLR--------------DDDIWNALTENYGN-VMPVDWKTSHTR :  99
Bf_FEZ2b     : ---------VVSFKSMEDLVHEFDETLNICFRNYNAK----------TDSIAPVKVLSQEEIME--------------NSELWQGLTDNFAN-VLPVDWNSSYTR : 124
Bf_FEZ2a     : ---------VVSFKSMEDLVHEFDETLNICFRNYNAK----------TDSIAPVKVLSQEEIME--------------NSELWQGLTDNFAN-VLPVDWNSSYTR : 118
Ci_FEZ1sim   : -------PELLSYSSMEDLVQTFDEKIAACFRINASNNNK--------VQENPVKPFNAETITD---------------DYIWKRLSDNYGL-VQPLNWETSLVR : 132
Ce_UNC76     : ------RLHDEFSGSLEDLVGNFDEKIAACLKDHEVT----------TADIAPVQIRTQEEVMN--------------ESQTWWTLTGNFGN-IQPLDFGTSSIC : 119
Bm_UNC76     : ------FDTENLSGSLEDLVGTFDQKISHCFKDLNKA----------TEEIAPIQVRSQDEIMS--------------ESQIWWTLTGNFGN-MPPLDFSKTQTR : 120
DPs_UNC76    : -------FTETFGGSLEDLVNTFDDKITKCFGNYEEN----------VEELAPVQVRSQEEIMN--------------ECQMWWTITGNFGN-ILPIDWSKSYTR : 194
Dp_UNC76     : -------FTETFGGSLEDLVNTFDDKITKCFGNYEEN----------VEELAPVQVRSQEEIMN--------------ECQMWWTITGNFGN-ILPIDWSKSYTR : 201
Ds_UNC76     : -------FTETFGGSLEDLVNTFDEKITKCFGNYEEN----------VEELAPVQVRSQEEIMN--------------ECQMWWTITGNFGN-ILPIDWSKSYTR : 201
Dm_UNC76     : -------FTETFGGSLEDLVNTFDEKITKCFGNYEEN----------VEELAPVQVRSQEEIMN--------------ECQMWWTITGNFGN-ILPIDWSKSYTR : 208
Dy_UNC76     : -------FTETFGGSLEDLVNTFDEKITKCFGNYEEN----------VEELAPVQVRSQEEIMN--------------ECQMWWTITGNFGN-ILPIDWSKSYTR : 210
Da_UNC76     : -------FTETFGGSLEDLVNTFDEKITKCFGNYEEN----------VEELAPVQVRSQEEIMN--------------ECQMWWTITGNFGN-ILPIDWSKSYTR : 206
Dw_UNC76     : -------FTETFGGSLEDLVNTFDEKITKCFGNYEEN----------VEELAPVQVRSQEEIMN--------------ECQMWWTITGNFGN-ILPIDWSKSYTR : 203
Dv_UNC76     : -------FTETFGGSLEDLVNTFDEKITKCFGNYEEN----------VEELAPVQVRSQEEIMN--------------ECQMWWTITGNFGN-ILPIDWSKSYTR : 194
Dmo_UNC76    : -------FTETFGGSLEDLVNTFDEKITKCFGNYEEN----------VEELAPVQVRSQEEIMN--------------ECQMWWTITGNFGN-ILPIDWSKSYTR : 212
Dg_UNC76     : -------FTETFGGSLEDLVNTFDEKITKCFGNYEEN----------VEELAPVQVRSQEEIMN--------------ECQMWWTITGNFGN-ILPIDWSKSYTR : 199
Tc_UNC76     : -------FTETFSGSLEDLVNTFDEKITKCFGNYEES----------VEKLAPVQVRTQEEIMN--------------ECQMWWTITGNFGN-ILPIDWSKSYAR : 165
Ag_UNC76     : ------DFAEAFTGSLEDLVNTFDEKITKCFGNYEQS----------VEELAPVQVRSQEEIMN--------------ECQMWWTITGNFGN-ILPIDWSKTYAR : 196
Nv_UNC76     : ------GSFSPFSGSLEDLVNTFDEKITTCFRDYGTN----------VESLAPVQVRSQEEIMN--------------ECQMWWTITGTFGN-ILPIDWSKSYAR : 142
Am_UNC76     : ------GTFSPFSGSLEDLVNTFDEKITSCFRDYGTN----------VESLAPVQVRTQEEIMN--------------ECQMWWTITGTFGN-ILPIDWSKSYAR : 143
Ph_UNC76     : -------FTETFSGSLEDLVNTFDERITKCFCDYEEN----------VEKLAPVQVRTQEEIMN--------------ECQMWWTITGNFGN-ILPIDWSKSYAR : 183
Ap_UNC76     : -------FGETFSGSLEDLVHTFDDKITRCFCDYEES----------VEKLAPVQIRTQEEIMN--------------ECQMWWTITGNFGN-ILPIDWSKSYAR : 183
```

```
                320         *       340         *       360         *       380         *       400         *       420
Pt_FEZ1     : --EALNGNCSDTEIHE-------------------------------------------------KEEE-EFNEKSEND----SGINEEP--------------- : 160
Hs_FEZ1     : --EALNGNCSDTEIHE-------------------------------------------------KEEE-EFNEKSEND----SGINEEP--------------- : 160
Mf_FEZ1     : --EALNGNCSDPEIHE-------------------------------------------------KEEE-EFNEKSEND----SGINEEP--------------- : 160
Mmt_FEZ1    : --EALNGNCSDTEIHE-------------------------------------------------KEEE-EFNEKSEND----SGINEEP--------------- : 160
Bt_FEZ1     : --EALNGNNSDTEIHE-------------------------------------------------KEEE-EFNEKSEHD----SGITEEP--------------- : 160
Cf_FEZ1     : --EALNGNSSDTEIHE-------------------------------------------------KEEE-EFNEKSEHD----SGINEEP--------------- : 160
Rn_FEZ1     : --EALNGNSSDTEIHE-------------------------------------------------KEEEDEFIEKSEND----SGINEEP--------------- : 161
Mm_FEZ1     : --EALNGNSSDIEIHE-------------------------------------------------KEEE-EFNEKSEND----SGINEEP--------------- : 160
Md_FEZ1     : --EALNGNISDTEIHE-------------------------------------------------KEEE-EFNEKSEND----SGINEEP--------------- : 160
Xt_FEZ1     : --ESLNGNMSDSEFHE-------------------------------------------------KDEE-ELNEKSEND----SGIIEEP--------------- : 159
Dr_FEZ1     : --ETLNGNLSDQEIHE-------------------------------------------------KDED-EMNEKNENA----NCLNEEP--------------- : 162
Ss_FEZ1     : --EGFNGNLSDQEIHE-------------------------------------------------KEEE-EMNEKNDNA----NCLSEEP--------------- : 169
Gg_FEZ1     : --EAPDG--TDPQLCE-------------------------------------------------KEDE-ELAERSEHD----SGINEEP--------------- : 147
Xt_FEZ2     : ALHLPTLNILDKAVNEN------------------------------------------LN-LDLSDDE-ELREQLDMHSIIVSCINEEP--------------- : 185
Xl_FEZ2     : ALHLPTLNILDKAVNDN------------------------------------------IN-LDLSDDE-ELREQLDMHSIIVSCINEEP--------------- : 158
Cf_FEZ2     : TLHLLTLNISEKGISDS------------------------------------------LL-FDTSDDE-ELREQLDMHSIIVSCVNEEP--------------- : 154
Bt_FEZ2     : TLHLLTLNISEKGMSDG------------------------------------------LL-FDTSDDE-ELREQLDMHSIIVSCVNDEP--------------- : 154
Hs_FEZ2     : TLHLLTLNISEKGVSDS------------------------------------------LL-FDTSDDE-ELREQLDMHSIIVSCVNDEP--------------- : 158
Pt_FEZ2     : TLHLLTLNISEKGVSDS------------------------------------------LL-FDTSDDE-ELREQLDMHSIIVSCVNDEP--------------- : 160
Rn_FEZ2     : TLHLLTLNISEKGMSDG------------------------------------------LP-FDTSDEE-ELREQLDMHSIIVSCVNEEP--------------- : 153
Mm_FEZ2     : TLHLLTLNISEKGMNDG------------------------------------------LL-FDASDEE-ELREQLDMHSIIVSCVNEEP--------------- : 157
Md_FEZ2     : TLHLLTLNISEKGINDN------------------------------------------LI-LDASDEE-ELREQLDMHSIIVSCINDEP--------------- : 152
Tg_FEZ2     : ALHLPTLNISEKGVNDN------------------------------------------LN-LDLSDDE-ELREQLDMHSIIVSCINEEP--------------- : 289
Ec_FEZ2     : TLHLLTLNISEKGISDS------------------------------------------LL-FDTSDDE-ELREQLDMHSIIVSCVDDEP--------------- : 164
Tn_FEZ2     : SLHLPILNISEQEKVDN------------------------------------------QS-LDLSDDE-ELREQMDMHSIIVSSISDEP--------------- : 172
Dr_FEZ2     : SLHLPTLNIRPQERLEV------------------------------------------NN-LDLSDDE-ELRDQMDMHTIIISCVNEEP--------------- : 145
Bf_FEZ2b    : QLYNKALNINEQK-REE------------------------------------------PMNLDLSDDE-ELRESFDAHSLIISSLTQEP--------------- : 170
Bf_FEZ2a    : QLYNKALNINEQKVREE------------------------------------------PMNLDLSDDE-ELRESFDAHSLIISSLTQEP--------------- : 165
Ci_FEZ1sim  : KLHIPALDIPGEKATSN------------------------------------------EKEVKAETEDPELEKQMLFHRMIEYNVYTEESGSVYQPPYGESNKM : 195
Ce_UNC76    : KKMAAALDSDSLKDD-----------------------------------------ASTRRSMTNSDDEDLIRQQMDVHQMIGHHHGSTD---------TGGETP : 174
Bm_UNC76    : RLQLPALDIQGPRKE-----------------------------------------NSD-LGIDLSEDEE-IRSALDMHQLISQRG-------------PLSESP : 169
DPs_UNC76   : QMHMPTLNIGQNHTKQ--QQQQQHQRNQHQLQKHQHSYPHTP--------------GGDEFN-DLASEDEAVANDLDMHALILNGLN--G---------DIDDQP : 271
Dp_UNC76    : QMHMPTLNIGQNHTKQ--QQQQQHQRNQHQLQKHQHSYPHTP--------------GGDEFN-DLASEDEAVANDLDMHALILNGLN--G---------DIDDQP : 278
Ds_UNC76    : QMHMPTLNIGQNHTKQ--QQQNRNQQQQLHTQSHQ-AYPHTN--GAGSGSGLDAQTPGDEFN-DLTSEDEAVANDLDMHALILNGLN--G---------DMDDQP : 289
Em_UNC76    : QMHMPTLNIGQNHTKQ--QQQNRNQQQQLHNQSHQ-AYPHTNGLGSGSGSGLDAQTPGDEFN-DLTSEDEAVANDLDMHALILNGLN--G---------DMDDQP : 298
Dy_UNC76    : QMHMPTLNIGQNHTKQ--QQQARNQQQQLHNQSHQ-AYPHTN----GSSAGLDAQTPGDEFN-DLTSEDEAVANDLDMHALILNGLN--G---------DMDDQP : 296
Da_UNC76    : QMHMPTLNIGQNPTKQ--QQNSRNQQQQNQLHHQSTHSNGFGPGSGSGSGSEAQTPGGDDFN-DLASEDEAVANDLDMHALILNGLN--G---------DLDDQP : 297
Dw_UNC76    : QMHMPTLNIGQSLTKQ--QQQQRNQQQQQQQQQQHYQQQHQQ------QQQHNAQTPSDEFN-DLTSEDEAVANDLDMHALILNGLNN-T---------DMDDQP : 289
Dv_UNC76    : QMHMPTLNIGQSRTKQ--QQQQRNQLQQQQQQQQNQQQQQSN--------TLDAQTPVDEFN-DLASEDEAVANDLDMHALILNGLN--G---------DADDHP : 277
Emo_UNC76   : QMHMPTLNIGQSRTKQ--QQQQQRNLQQQQQQHQQQNQQ----------------TPVDEFN-DLTSEDEAVANDLDMHALILNGLN--G---------DADDHP : 287
Dg_UNC76    : QMHMPTLNIGQNRTKQRSQQQQQQQQQQQQQQQSPHKQQQQQ--------QQNAQTPVDEFN-DLASEDEAVANDLDMHALILNGLN--G---------DVD-EP : 283
Tc_UNC76    : KLHMPALNINEKEATT----------------------------------------PDDDL--ELSSEDEAVASDLDMHALILGGLH-------------QDTEP : 215
Ag_UNC76    : QMHVPALNIGMRKPGT----------------------------------------PDDELLQDLSSEDEAVASDLDMHALILGGLH-------------ADNEP : 248
Nv_UNC76    : KLHVPTLNINESAKSF----------------------------------------DGNGLE-DLSSEDEAVATDLDMHALILSSRND-N---------YNVEEP : 196
Am_UNC76    : KMHMPALNINEAPVSQ----------------------------------------ERPELE-DLSSEDEAVATDLDMHALILSSSTD-T---------HSPEEP : 197
Ph_UNC76    : KLHVPTLNITENNGGN----------------------------------------SNDDII-NLSDEDEQVANDLDMHSLILNGLR-------------QDCES : 234
Ap_UNC76    : KVQLPVLNINNNIQDE----------------------------------------SSD----DLSSEDEAVSNDLDLHTLILSGPS-------------ADIEP : 231
```

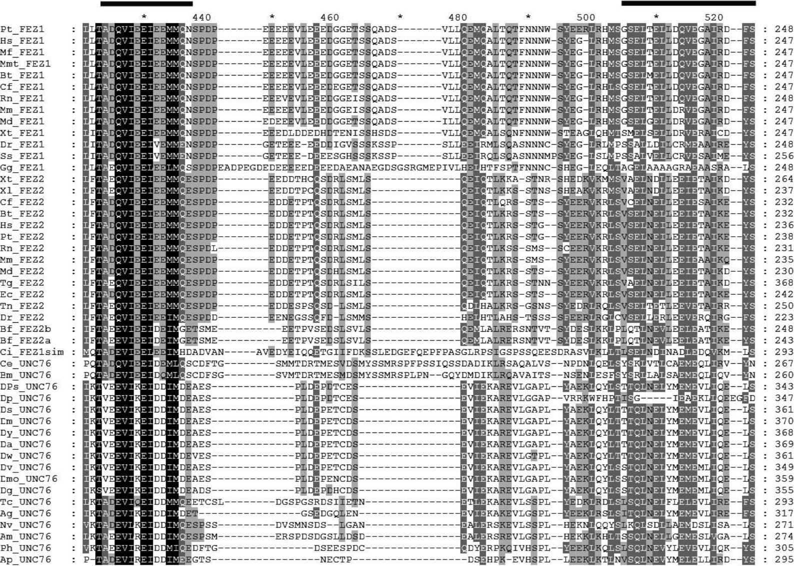

* 440 * 460 * 480 * 500 * 520
Pt_FEZ1 : ILTADQVIEEIEEMMQNSPDP-------EEEEEVLEEEDGGETSSQADS-------VLLQEMQALTQTFNNNW-SYEERLRHMSGSELTELLDQVEGAIRD--FS : 248
Hs_FEZ1 : ILTADQVIEEIEEMMQNSPDP-------EEEEEVLEEEDGGETSSQADS-------VLLQEMQALTQTFNNNW-SYEG-LRHMSGSELTELLDQVEGAIRD--FS : 247
Mf_FEZ1 : ILTADQVIEEIEEMMQNSPDP-------EEEEEVLEEEDGGETSSQADS-------VLLQEMQALTQTFNNNW-SYEG-LRHMSGSELTELLDQVEGAIRD--FS : 247
Mmt_FEZ1 : ILTADQVIEEIEEMMQNSPDP-------EEEEEVLEEEDGGETSSQADS-------VLLQEMQALTQTFNNNW-SYEG-LRHMSGSELTELLDQVEGAIRD--FS : 247
Bt_FEZ1 : ILTADQVIEEIEEMMQNSPDP-------EEEEEVLEEEDGGETSSQADS-------VLLQEMQALTQTFNNNW-SYEG-LRHMSGSELMELLDQVEGAIRD--FS : 247
Cf_FEZ1 : ILTADQVIEEIEEMMQNSPDP-------EEEEEVLEEEDGGEGTSSQADS-------VLLQEMQALTQTFNNNW-SYEG-LRHLSGSELTELLDQVEGAIRD--FS : 247
Rn_FEZ1 : ILTADQVIEEIEEMMQNSPDP-------EEEVEVLEEEDGGEISSQADS-------VLLQEMQALTQTFNNNW-SYEG-LRHMSGSELTELLDQVEGAIRD--FS : 248
Mm_FEZ1 : ILTADQVIEEIEEMMQNSPDP-------EEEEEVLEEEDGGEISSQADS-------VLLQEMQALTQTFNNNW-SYEG-LRHMSGSELTELLDRVEGAIRD--FS : 247
Md_FEZ1 : ILTADQVIEEIEEMMQNSPDP-------EDEEEVLEEDDGGETSSQADS-------ILLQEMQALTQTFNNNW-SYEG-LRHMSGSELGELLDRVEGAIRD--YS : 247
Xt_FEZ1 : ILTADQVIEEIEEMMQNSPDP-------EEEDLDDEDHDTENISSHSDS-------VLLQEMQALSQNFNNNW-STEAGLQHMTSMELSELLDRVERAICD--YS : 247
Dr_FEZ1 : ILTADQVIEEIVEMMENSPDP-------GETEEE-EEDDIGVSSSKSSP-------SLLEEIRMLSQASNNNC-SYEG-LRLMPSSALLDLLCRMEAAIRE--YS : 248
Ss_FEZ1 : ILTADQVIEEIVEMMENSPDP-------GETEEE-DEESGHSSSKSSP-------SLLEEIRQLSQASNNNMPSYEG-LSLMPSSALVELLCRVESALME--YS : 256
Gg_FEZ1 : ILTAEQVIEELEELMQSSPDPEADPEGDEDEEEEEDEEEDAEANAEGDSGSRGMEPIVLHELHTFSPTFNNNC-SHEG-LEQLTAGELAAAAGRAEAASRA--LS : 248
Xt_FEZ2 : IFTAEQVIEEIEEMMQESPDP--------EEDDTHCQSDRLSMLS-------------------QEICTLKKA-STNR-SHEDKVKMMSVAEINDLLEEIETAIKD--YS : 264
Xl_FEZ2 : IFTAEQVIEEIEEMMQESPDP--------EEDDTPCQSDRLSMLS-------------------QEICTLKKS-STNS-SHEAKVKRMSVAEINDLLEEIETAIKD--YS : 237
Cf_FEZ2 : IFTADQVIEEIEEMMQESPDP--------EDDETPTQSDRLSMLS-------------------QEICTLQRS-STS--SYEERVKRLSVCEINEILEEIESAIKE--YS : 232
Bt_FEZ2 : IFTADQVIEEIEEMMQESPDP--------EDDETPTQSDRLSMLS-------------------QEICTLKRS-STS--SYEERVKRLSVSEINEILEEIETAIKE--YS : 232
Hs_FEZ2 : IFTADQVIEEIEEMMQESPDP--------EDDETPTQSDRLSMLS-------------------QEICTLKRS-STG--SYEERVKRLSVSEINEILEEIETAIKE--YS : 236
Pt_FEZ2 : IFTADQVIEEIEEMMQESPDP--------EDDETPTQSDRLSMLS-------------------QEICTLKRS-STG--SYEERVKRLSVSEINEILEEIETAIKE--YS : 238
Rn_FEZ2 : IFTADQVIEEIEEMMQESPDL--------EDDETPTQSDRLSMLS-------------------QEICTLKSS-SMS--SCEERVKRLSVSEINEILEEIETAIKE--YS : 231
Mm_FEZ2 : IFTADQVIEEIEEMMQESPDP--------EDDETPTQSDRLSMLS-------------------QEICTLKRS-SMS--SYEERVKRLSVSEINEILEEIEAAIKQ--YS : 235
Md_FEZ2 : IFTADQVIEEIEEMMQESPDP--------EDDETPTQSDHLSMLS-------------------QEICTLKRS-STS--SYEERVKRLSVSEINEILEEIETAIKD--YS : 230
Tg_FEZ2 : IFTAEQVIEEIEEMMQESPDP--------EDDETPTQSDRLSILS-------------------QEICTLKRS-STNN-SYEERVKRLSVAEINEILEEIETAIKD--YS : 368
Ec_FEZ2 : IFTADQVIEEIEEMMQESPDP--------EDDETPTQSDRLSMLS-------------------QEICTLKRS-STS--SYEERVKRLSVSEINEILEEIETAIKE--YS : 242
Tn_FEZ2 : IFTAEQVIEEIEEMMQESPDP--------EDDESPSQSD-LSMLS-------------------QDIHALKRS-GSNS-SYEDRIRQLSVSEITETLEEVETAIRR--YS : 250
Dr_FEZ2 : IFTAEQVIEEIEEIMQESPDD--------EENEGSSQFD-LSMMS-------------------HEIHTLAHS-TSSS-SEERIRGICVSEILERLEEVERQIRG--FS : 223
Bf_FEZ2b : IFTAEEVIEEIDEIMGETSME----------EETPVSEDSLSVLS-------------------QEMLALRERSNTVT-SYDESLKKIPLQTINEVLEELEATIKE--YS : 248
Bf_FEZ2a : IFTAEEVIEEIDEIMGETSME----------EETPVSEDSLSVLS-------------------QEMLALRERSNTVT-SYDESLKKIPLQTINEVLEELEATIKE--YS : 243
Ci_FEZ1sim : MQTADEVIEELEEIMHDADVAN-----AVEDYEIQQETGIIFDKSSLEDGEFQEPFPASGLRPSIGSPSSQEESDRASVLKLLTLSEINDINADLEDQVKM--LS : 293
Ce_UNC76 : PQTADQVIEEIDEMLQSCDFTG--------SMMTDRTMESVDSMYSSMRSPFPSSIQSSDADIKLRSAQALVS--NPDNLQELSYSKIVTICAEMEQLIRV--YN : 267
Bm_UNC76 : PQTADEVIEEIDQMLQSCDFSG--------SVMTDRTMESMDSMYSSMRSPLPN--GQYDMDIKLRQAVAITS--NSENLESFSYSRILALSAEMEQLIQV--YN : 260
DPs_UNC76 : IKTVEEVIKEIDDIMDEAES------------------PLDEPDTCES-----------------EVIEKAREVLGAPL--YAEKLQYLSTTQINELYMEMEVLIQE--LS : 343
Dp_UNC76 : IKTVEEVIKEIDDIMDEAES------------------PLDEPDTCES-----------------EVIEKAREVLGGAP--VRRKWFHPTISG-----IEAEKLLQEEGED : 347
Ds_UNC76 : IKTVEEVIKEIDDIMDEAES------------------PLDEPETCDS-----------------EVIEKAREVLGAPL--YAEKLQYLTTTQINELYMEMEVLIQE--LS : 361
Dm_UNC76 : IKTVEEVIKEIDDIMDEAES------------------PLDEPETCDS-----------------EVIEKAREVLGAPL--YAEKLQYLTTTQINELYMEMEVLIQE--LS : 370
Dy_UNC76 : IKTVEEVIKEIDDIMDEAES------------------PLDEPETCDS-----------------EVIEKAREVLGAPL--YAEKLQYLTTTQINELYMEMEVLIQE--LS : 368
Da_UNC76 : IKTVEEVIKEIDDIMDEAES------------------PLDEPETCDS-----------------EVIEKAREVLGAPL--YAEKLQYLTTTQINELYMEMEVLIQE--LS : 369
Dw_UNC76 : IKTVEEVIKEIDDIMDEVES------------------PLDEPETCDS-----------------EVIEKAREVLGTPL--YAEKLQYLTTTQINELYMEMEVLIQE--LS : 361
Dv_UNC76 : IKTVEEVIKEIDDIMDEAES------------------PLDEPETCDS-----------------EVIEKAREVLGAPL--YAEKLQYLSSTQINELYMEMEMLIQE--LS : 349
Dmo_UNC76 : IKTVEEVIKEIDDIMDEAES------------------PLDEPENCDS-----------------EVIEKAREVLGAPL--YAEKLQYLSSTQINELYMEMEMLIQE--LS : 359
Dg_UNC76 : IKSVEEVIKEIDDIMDEADS------------------PLDEPDHCDS-----------------EVIEKAREVLGAPL--YAEKLQYLSSTQINELYMEMEMLIQE--LS : 355
Tc_UNC76 : IKTADEVIQEIDDMMQEETCSL--------DGSPGSRDSIIETN-----------------ETLEKAKEVLSSPL--YEDKLRDLSLSQINELFLELEVLIRE--FS : 293
Ag_UNC76 : IKTADEVIKEIDDIMDET--------------------GSEDGQLEN-----------------EVIEKAKEVLGSPL--YEEKLRSLSITQINELYMEMEVLIRE--FS : 317
Nv_UNC76 : VKTADEVIKEIDDIMQESPSS---------DVSMNSDS--LGAN-----------------EALERSKEVLSSPL--HEKNLQQYSLKQISDILAEMDSLISA--LS : 271
Am_UNC76 : IKTAEEVIREIDDIMQESPSM---------ERSPDSDGSLLDSD-----------------EALERSKEVLGSPL--HEKKLKHLTSSQINELLGEMESLVGA--LS : 274
Ph_UNC76 : VKTAEEVIREIDDMIQEDFTG-----------------DSEESPDC-----------------QDYERPKQIVHSPL--YEEKLQSLSLSQINEVYLEVEVLIQK--YS : 305
Ap_UNC76 : P-TADEVIREIDDIMEEGTS----------------------NECTP-----------------DSEHPK-EVHSPL--LEEKLKTLNVSQINEVYMELELLIRD--YS : 295

```
                   *        540          *        560          *        580          *        600          *        620          *
Pt_FEZ1    : EELVQQLARRDELEFEKEVKNSFITVLIEVQNKQKEQRELMKKRRK-EKGLSLQS------------------SRIEKGNQMPLK--------------------- : 314
Hs_FEZ1    : EELVQQLARRDELEFEKEVKNSFITVLIEVQNKQKEQRELMKKRRK-EKGLSLQS------------------SRIEKGNQMPLKRFSMEGISNILQSGIRQTFGS : 334
Mf_FEZ1    : GELVQQLARRDELEFEKEVKNSFITVLIEVQNKQKEQRELMKKRRK-EKGLSLQS------------------SRIEKGNQMPLKRFSMEGISNILQSGIRQTFGS : 334
Mmt_FEZ1   : EELVQQLARRDELEFEKEVKNSFITVLIEVQNKQKEQRELMKKRRK-EKGLSLQS------------------SRIEKGNQMPLKRFSMEGISNILQSGIRQTFGS : 334
Bt_FEZ1    : EELVQQLARRDELEFEKEVKNSFITVLIEVQNKQKEQRELMKKRRK-EKGLSLQS------------------SRIDKGSQMPLKRFSMEGISNILQSGIRQTFGP : 334
Cf_FEZ1    : EELVQQLARRDELEFEKEVKNSFITVLIEVQNKQKEQRELMKKRRK-EKGLSLQS------------------SRIEKGSQMPLKRFSVEGISNILQSGIRQTFGS : 334
Rn_FEZ1    : EELVHQLARRDELEFEKEVKNSFITVLIEVQNKQKEQRELMKKRRK-EKGLSLQS------------------SRIEKGSQMPLKRFSMEGISNILQSGIRQTFGS : 335
Mm_FEZ1    : EELVHQLARRDELEFEKEVKNSFITVLIEVQNKQKEQRELMKKRRK-EKGLSLQS------------------NRIEKGSQMPLKRFSMEGISNILQSGIRQTFGS : 334
Md_FEZ1    : EELVQQLARRDELEFEKEVKNSFITVLIEVQNKQKEQRELMKKRRK-EKGLSLQS------------------SRIEKGNQMPLKRFSMEGISNILQTGIRQTFGN : 334
Xt_FEZ1    : EELVQQLARRDELEFEKEVKNSFITALIEVQNKQKEHRELVKKRRK-DKGMSLHI------------------SRAENNGQMPMKRFSIEGLSNILQSGIRQTFGN : 334
Dr_FEZ1    : EELVAQLARRDELEFEKEVKNTFITALMEVQNRQKEQRELSKRRRK-DKAMSLQG-----------------STRPEKTGSMPAKRFSMEGLSNILQTGIRQTFGS : 336
Ss_FEZ1    : EELVTQLARRDELEFEKEVKNSFITALMDVQNRQKEQRDTGKRRRRSDKGLSLQGNAVTTTVVGGTTTTTNAPTDSKPASMPMKRFSMEGLSNILQTGIRQTFGS : 361
Gg_FEZ1    : AELVAQLARRDELAFEKEVKTAFIGALIAVQGEQREQREAARRRRR-DRGLSMQG-----------------GRPERGGHMPRK----------------------- : 314
Xt_FEZ2    : EELIQQLALRDELEFEKEVKNSFISVLIEVQNKQKEHKENVKKKRRVKS---ANA-----------------QNGTKERSHMPGT--------------------- : 329
Xl_FEZ2    : EELIQQLALRDELEFEKEVKNSFISVLIEVQNKQKEHKENVKKKRKLKS---ANA-----------------QNGTKERSHMPGT--------------------- : 302
Cf_FEZ2    : EELVQQLALRDELEFEKEVKNSFISVLIEVQNKQKEHKETAKKKKKLKN---GSS-----------------QNGKNERSHMPGT--------------------- : 297
Bt_FEZ2    : EELVQQLALRDELEFEKEVKNSFISVLIEVQNKQKEHKETAKKKKKLKN---GSS-----------------QNGKNERSHMPGTRFSMEGISNVIQNGLRHTFGN : 318
Hs_FEZ2    : EELVQQLALRDELEFEKEVKNSFISVLIEVQNKQKEHKETAKKKKKLKN---GSS-----------------QNGKNERSHMPGT--------------------- : 301
Pt_FEZ2    : EELVQQLALRDELEFEKEVKNSFISVLIEVQNKQKEHKETAKKKKKLKN---GSS-----------------QNGKNERSHMPGT--------------------- : 303
Rn_FEZ2    : EELVQQLALRDELEFEKEVENSFISALIEVQNKQKEHKETAKKKKKLKN---GSS-----------------QNGRNERSHMPGT--------------------- : 296
Mm_FEZ2    : EELVQQLALRDELEFEKEVENSFISALIEVQNKQKEHKETAKKKKKLKS---GSS-----------------QNGRSERSHMPGT--------------------- : 300
Md_FEZ2    : EELVQQLALRDELEFEKEVKNSFISVLIEVQNKQKEHKETAKKKKKLKN---SSP-----------------QNGKNEKSHMPGT--------------------- : 295
Tg_FEZ2    : EELVQQLALRDELEFEKEVKNSFISVLIEVQNKQREHKETAKKKKKLKN---GSP-----------------QNGKQERGHMPGTRFSMEGISNVIQNGFRHTFGN : 454
Ec_FEZ2    : EELVQQLALRDELEFEKEVKNSFISVLIEVQNKQKEHKETAKKKKKLKN---GGS-----------------QNGKNERGHMPGTRFSMEGISNVIQNGLRHTFGN : 328
Tn_FEZ2    : EELIQALALRDELDYEKEVKNSFISLLIDVQNRQKEHRELLRKKKKIRSTVTTGP-----------------NGQRTTSTHIPGTLLTLEGLSNVIHNGLRQTFGS : 339
Dr_FEZ2    : EELIDQLAVREELDYEKEVKNTFISALIHVQNRQKEHRELLKKKRKIKS---TG------------------GVQRSDRSHVPG---------------------- : 286
Bf_FEZ2b   : EILIQQLALRDELVYEKEVKNQFISALVAVQNRQREHKQNKDNKKKRRK---------------------VKGGNGTEPGT------------------------- : 308
Bf_FEZ2a   : EILIQQLALRDELVYEKEVKNQFISALVAVQNRQREHKQNKDNKKKRRK---------------------VKGGNGTEPGT------------------------- : 303
Ci_FEZ1sim : ADLIHEIGRRDELNYENEVKNCFISRVIEVQYKQEQFRKVAAAPPKKFG-----------------RLRKSATTDSVSAG-------------------------- : 356
Ce_UNC76   : ESLVDELAHRDELDYEKEMKNSFISLLIAIQNKRRVYANDRKRKVGKASDASQ------------------------------------------------------ : 320
Bm_UNC76   : DSLVEQLAHRDELEYEKEMKNTFISLLISIQNRRRHFTNERKRKP-LKTDPSQ------------------------------------------------------ : 312
DPs_UNC76  : ETLINELALRDELEYEKELKNSFISLLIAVQNKRRQYHVEKKRGKFQGPE--------------------------------------------------------- : 393
Dp_UNC76   : GSFLARLSSSNPGAFTLSVRRGNEVTHIKIQNNGDFFDL------YGGEK--------------------------------------------------------- : 391
Ds_UNC76   : ETLINELALRDELEFEKELKNSFISLLIAVQNKRRQYHVEKKRGKFQGPE--------------------------------------------------------- : 411
Dm_UNC76   : ETLINELALRDELEFEKELKNSFISLLIAVQNKRRQYHVEKKRGKFQGPE--------------------------------------------------------- : 420
Dy_UNC76   : ETLINELALRDELEFEKELKNSFISLLIAVQNKRRQYHVEKKRGKFQGPE--------------------------------------------------------- : 418
Da_UNC76   : ETLINELALRDELEFEKELKNSFISLLIAVQNKRRQYHVEKKRGKFQGPE--------------------------------------------------------- : 419
Dw_UNC76   : ETLINELALRDELEFEKELKNSFISLLIAVQNKRRQYHVEKKRGKFQGPE--------------------------------------------------------- : 411
Dv_UNC76   : ETLINELALRDELEYEKELKNSFISLLIAVQNKRRQYHVEKKRGKFQGPE--------------------------------------------------------- : 399
Dmo_UNC76  : ETLINELALRDELEYEKELKNSFISLLIAVQNKRRQYHVEKKRGKFQGPE--------------------------------------------------------- : 409
Dg_UNC76   : ETLINELALRDELEYEKELKNSFISLLIAVQNKRRQYHVEKKRGKFQGPE--------------------------------------------------------- : 405
Tc_UNC76   : ETLISELALRDELEYEKELKNTFISLLIAVQNKRRQYHVEKKRSLKNASKTPSGL---------------------------------------------------- : 348
Ag_UNC76   : ETLISELALRDELEYEKELKNTFISLLIAVQNKRRQYHVEKKG-KAGGKGSAGM----------------------------------------------------- : 371
Nv_UNC76   : ETLISELALRDELEYEKELKNQFISLLIAVQNRRRQHHVAKKRNQLQNGTSPLLQRK-------------------------------------------------- : 328
Am_UNC76   : ETLIAELALRDELEYEKELKNQFISLLIAVQNRRRQHHVTKKRNQMQNGTSPLLQHR-------------------------------------------------- : 331
Ph_UNC76   : ETLISELALRDELEYEKELKNTFIDLLVSVQNRRRQYHAERKRCNRNGNSPRTNAV--------------------------------------------------- : 361
Ap_UNC76   : ETLITELALRDELEFEKELKNTFISLLIAVQNRRRQYHVEKKRNSRAGNTNKNVTN--------------------------------------------------- : 351
```

```
                    640         *       660         *       680         *       700         *       720         *
Pt_FEZ1    : ------YLNTVIPYEKKASPPSVEDLQMLTN----------------------------ILFAMKEDNEKVPTLLTDYILK-VLCPT------------------ : 366
Hs_FEZ1    : SGTDKQYLNTVIPYEKKASPPSVEDLQMLTN----------------------------ILFAMKEDNEKVPTLLTDYILK-VLCPT------------------ : 392
Mf_FEZ1    : SGSDKQYLNTVIPYEKKASPPSVEDLQMLTN----------------------------ILFAMKEDNEKVPTLLTDYILK-VLCPT------------------ : 392
Mmt_FEZ1   : SGSDKQYLNTVIPYEKKASPPSVEDLQMLTN----------------------------ILFAMKEDNEKVPTLLTDYILK-VLCPT------------------ : 392
Bt_FEZ1    : SGSDKQYLNTVIPYEKKASPPSVEDLQMLTN----------------------------ILFAMKEDNEKVPTLLTDYILK-VLCPT------------------ : 392
Cf_FEZ1    : SGTDKQYLNTVIPYEKKALPPSVEDLQMLTN----------------------------ILFAMKEDNEKVPTLLTDYILK-VLCPT------------------ : 392
Rn_FEZ1    : SGADRQYLNTVIPYEKKSSPPSVEDLQMLTN----------------------------ILFAMKEDNEKVPTLLTDYILK-VLCPT------------------ : 393
Mm_FEZ1    : SGADRQYLNTVIPYEKKSSPPSVEDLQMLTN----------------------------ILFAMKEDNEKVPTLLTDYILK-VLCPT------------------ : 392
Md_FEZ1    : SGTDKQYLNTVIPYEKKASPPSVEDLQMLTN----------------------------ILFAMKEDNEKVPTLLTDYILK-VLCPT------------------ : 392
Xt_FEZ1    : SGNDKQYLNTVIPYEKRGTPPSVEDLQMLTN----------------------------ILYAMKEDSEKVPMLLTDYILK-VLCPT------------------ : 392
Dr_FEZ1    : SGTEKQYLNTVIPYEKKGTPPSVDDLQMLTK----------------------------ILYAMKEDSEKVPTLLTDYILK-VLCPT------------------ : 394
Ss_FEZ1    : TGTDKQYLNTVIPYEKKATPPTVDDLQMLTN----------------------------ILYAMKEDSEKVPTLLTDYILK-VLCPT------------------ : 419
Gg_FEZ1    : ------YLNTVIPYEKKGSPPSVEDLQMLTN----------------------------ILFAMKEGNEKVPTLLTDYILK-VLCPT------------------ : 366
Xt_FEZ2    : ------YLTTVIPYEEKNGPPSIEDLQILTK----------------------------ILRAMKDDSDKVPSLLTDYILK-VLCPT------------------ : 381
Xl_FEZ2    : ------YLTTVIPYEEKNGPPSVEDLQILTK----------------------------ILHAMKDDSDKVPSLLTDYILK-VLCPT------------------ : 354
Cf_FEZ2    : ------YLTTVIPYEKKNGPPSVEDLQILTK----------------------------ILRAMKEDSEKVPSLLTDYILK-VLCPT------------------ : 349
Bt_FEZ2    : SGGEKQYLTTVIPYEKKNGPPSVEDLQILTK----------------------------ILRAMKEDSEKVPSLLTDYILK-VLCPT------------------ : 376
Hs_FEZ2    : ------YLTTVIPYEKKNGPPSVEDLQILTK----------------------------ILCAMKEDSEKVPSLLTDYILK-VLCPT------------------ : 353
Pt_FEZ2    : ------YLTTVIPYEKKNGPPSVEDLQILTKKRRLLVNNPIPLFLSPLFGNFKSGKQLSVLRAMKEDSEKVPSLLTDYILK-VLCPT------------------ : 383
Rn_FEZ2    : ------YLTTVIPYEKKNGPPSVEDLQILTK----------------------------ILHAMKEDSEKVPSLLTDYILK-VLCPT------------------ : 348
Mm_FEZ2    : ------YLTTVIPYEKKSGPPSVEDLQILTK----------------------------ILHAMKEDSEKVPSLLTDYILK-VLCPT------------------ : 352
Md_FEZ2    : ------YLTTVIPYEKKNGPPSVEDLQILTK----------------------------ILHAMKEDSDKVPSLLTDYILK-VLCPT------------------ : 347
Tg_FEZ2    : SSGEKQYLTTVIPYEKKNGPPSVEDLQTLTK----------------------------ILHAMKEDSEKVPSLLTDYILK-VLCPT------------------ : 512
Ec_FEZ2    : SGAEKQYLTTVIPYEKKNGPPSVEDLQILTK----------------------------ILHAMKEDSEKVPSLLTDYILKGKQCAPNCAPHVRAAGSSL----- : 400
Tn_FEZ2    : TGGDKQYLTTVIPYEKKAGTPSVEDLQILTK----------------------------ILHAMRDDSEKVPALLTDYILK-GKPAAATFF-------------- : 401
Dr_FEZ2    : -----TYLTTVIPYDRSSGSPSVDDLQILTK----------------------------ILHAMREDSDTVPALLTDYILK-VLCPT------------------ : 339
Bf_FEZ2b   : ------FLYTVIPYEKKNSPMTAEDLQILTK----------------------------ILVAMSEDSDKVPSLLTDYILKGPQLDSETPFNWIKHIKLAVHSII : 379
Bf_FEZ2a   : ------FLYTVIPYEKKNSPMTAEDLQILTK----------------------------ILVAMSEDSDKVPSLLTDYILK------------------------ : 350
Ci_FEZ1sim : -----RYLTSVIPCS-SGPTPTLENLQAMIK----------------------------ILDAIKSDSDEVPSLLTAYILK-VLCPAPNGSRTLKL--------- : 417
Ce_UNC76   : ---LPQYLTATIPYND-HQHIDNASIASLIK----------------------------ILRAIHDDNTTVPTLLTDYILT-HVCPKNISC-------------- : 378
Bm_UNC76   : ---LPQYMTATIPYDESCLYVDMNTIMALIK----------------------------LLRAIDEDSPAVPSMLTDYILT-VLCPSASSSVITDLAA------- : 378
DPs_UNC76  : ----PKYLTTVIPYHLENGTPNNQSLQVLIK----------------------------ILKAINEDSPTVPALLTDYILK-VLCPT------------------ : 447
Dp_UNC76   : ----FATLPELVQYYMENGELKEKNGQAIEL----------------------------KQPLICAEPTTER--------------------------------- : 431
Ds_UNC76   : ----PKYLTTVIPYHLENGTPNNQSLQVLIK----------------------------ILKAINEDSPTVPALLTDYILK-VLCPT------------------ : 465
Dm_UNC76   : ----PKYLTTVIPYHLENGTPNNQSLQVLIK----------------------------ILKAINEDSPTVPALLTDYILK-VLCPT------------------ : 474
Dy_UNC76   : ----PKYLTTVIPYHLENGTPNNQSLQVLIK----------------------------ILKAINEDSPTVPALLTDYILK-VLCPT------------------ : 472
Da_UNC76   : ----PKYLTTVIPYHLENGTPNNQSLQVLIK----------------------------ILKAINEDSPTVPALLTDYILK-VLCPT------------------ : 473
Dw_UNC76   : ----PKYLTTVIPYHLENGTPNNQSLQILIK----------------------------ILKAINEDSPTVPALLTDYILK-VLCPT------------------ : 465
Dv_UNC76   : ----PKYLTTVIPYHLENGTPNNQSLQVLIK----------------------------ILKAINEDSPTVPTLLTDYILK-VLCPT------------------ : 453
Dmo_UNC76  : ----PKYLTTVIPYHLENGTPNNQSLQVLIK----------------------------ILKAINEDSPTVPTLLTDYILK-VLCPT------------------ : 463
Dg_UNC76   : ----PKYLTTVIPYHLENGTPNNQSLQVLIK----------------------------ILKAINEDSPTVPTLLTDYILK-VLCPT------------------ : 459
Tc_UNC76   : ---DPKYLTTVIPYHLDSGPPDNQTLQVLIK----------------------------ILKAINEDSPTVPTLLTDYILK-VICPT------------------ : 403
Ag_UNC76   : ---DPKYLTTVIPYQLNS-TPDNQTLQVLIK----------------------------ILKAINEDSPTVPTLLTDYILK-VLCPT------------------ : 425
Nv_UNC76   : SIQESKYLTTVIPYHTDNGLLDTQALQVLIK----------------------------ILKAINEDSPAVPALLTDYILK-VLCPT------------------ : 386
Am_UNC76   : SLQESKYLTTVIPYHTDSGPPDNQALQVLIK----------------------------ILKAISEDSPTVPTLLTDYILK-VLCPT------------------ : 389
Ph_UNC76   : ---ESKYLTTVIPYHKDSGPPNNQALQVLIK----------------------------ILKAIKEDNPMVPTLLTDYILK-VLCPT------------------ : 416
Ap_UNC76   : ---EPKYLTTVIPYHLDSGPLSNQALQVLIK----------------------------ILKAINEDSPTVPTLLTDYILK-EINIMFLSYSLILL--------- : 415
```
