# Supplementary figures and images for: FEZ2 Has Acquired Additional Protein Interaction Partners Relative to FEZ1: Functional and Evolutionary Implications
Source: PLoS One. 2011 Mar 8;6(3):e17426. doi: 10.1371/journal.pone.0017426 (PMC3050892; doi:10.1371/journal.pone.0017426)

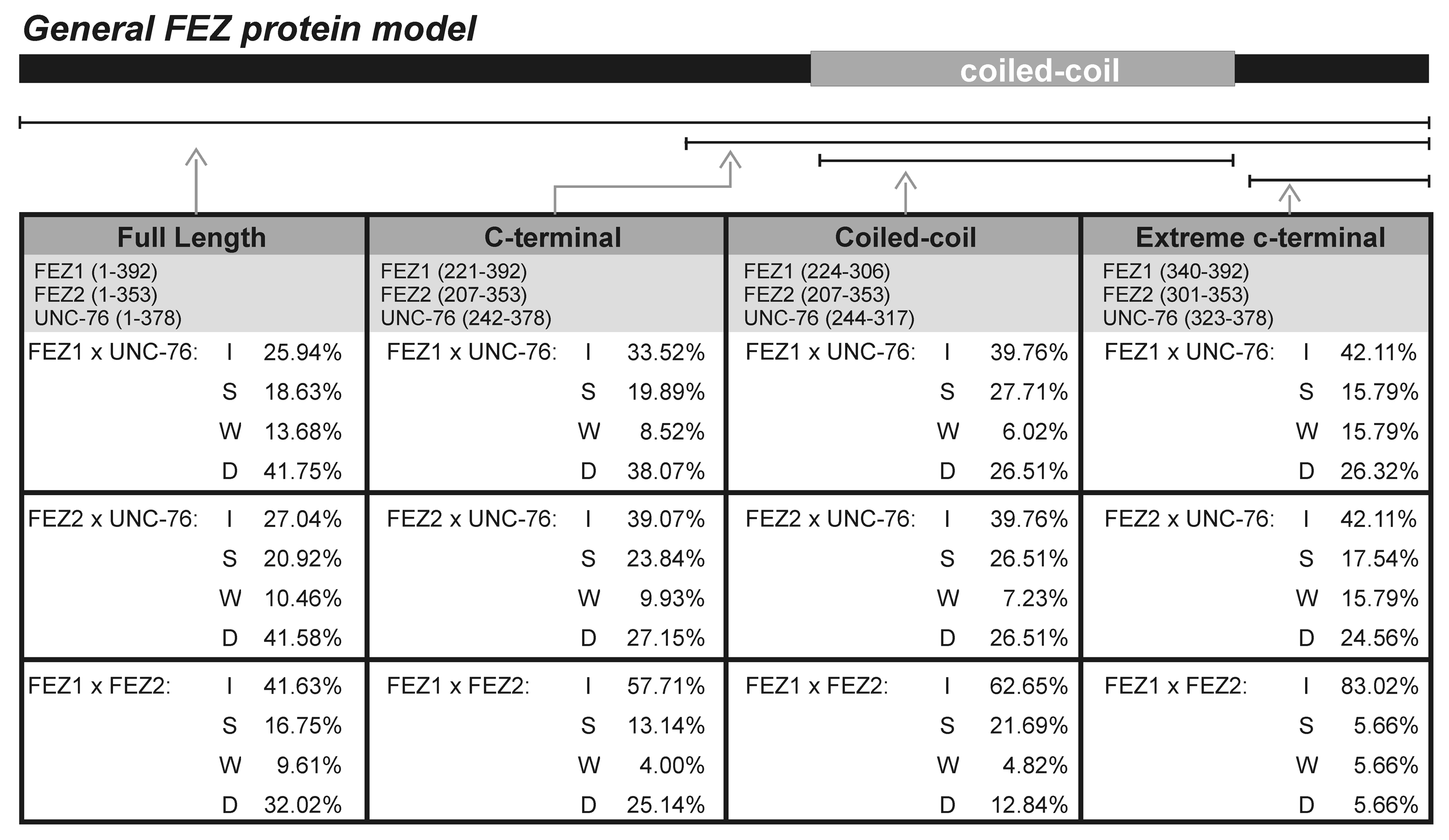

Supplement: Figure S2 — Identities and similarities between human proteins FEZ1 and FEZ2 and C. elegans UNC-76. A general scheme of the FEZ family proteins is shown at the top. The identity and similarity comparisons were made of two-by-two proteins both for the complete protein alignment as well as for local alignment of FEZ fragments by NPS@ (http://npsa-pbil.ibcp.fr/cgi-bin/npsa_automat.pl?page=npsa_clustalw.html). I = identity, S = strongly similar, W = weakly similar, D = different. (TIF) [file pone.0017426.s002.tif]
