# Supplementary material for: FEZ2 Has Acquired Additional Protein Interaction Partners Relative to FEZ1: Functional and Evolutionary Implications
Source: PLoS One. 2011 Mar 8;6(3):e17426. doi: 10.1371/journal.pone.0017426 (PMC3050892; doi:10.1371/journal.pone.0017426)
Supplement: Table S1 — FEZ family proteins used for amino acid sequence alignment. (DOC) [file pone.0017426.s003.doc]

**Table S1: FEZ family proteins used for amino acid sequence alignment**

| **Number** | **Name** | **Identifier** | **Organism** | **Taxonomy** | **Description** |
| --- | --- | --- | --- | --- | --- |
| 1 | Cf_FEZ2 | gi|73980130| | Canis familiaris | Eu., mt., vt., mam. | PREDICTED: similar to FEZ2 |
| 2 | Hs_FEZ2 | gi|114205570| | Homo sapiens | Eu., mt., vt., mam. | Fez2 protein |
| 3 | Bt_FEZ2 | gi|76629046| | Bos taurus | Eu., mt., vt., mam. | PREDICTED: similar to FEZ2 |
| 4 | Rn_FEZ2 | gi|72679487| | Rattus norvegicus | Eu., mt., vt., mam. | Fez2 protein |
| 5 | Pt_FEZ2 | gi|114576932| | Pan troglodytes | Eu., mt., vt., mam. | PREDICTED: FEZ2 |
| 6 | Md_FEZ2 | gi|126303164| | Monodelphis domestica | Eu., mt., vt., mam. | PREDICTED: FEZ2 |
| 7 | Mm_FEZ2 | gi|148706509| | Mus musculus | Eu., mt., vt., mam. | Fez2 protein |
| 8 | Md_FEZ1 | gi|126327241| | Monodelphis domestica | Eu., mt., vt., mam. | PREDICTED: hypothetical protein |
| 9 | Mf_FEZ1 | gi|67970547| | Macaca fascicularis | Eu., mt., vt., mam. | unnamed protein product |
| 10 | Bt_FEZ1 | gi|66792834| | Bos taurus | Eu., mt., vt., mam. | zygin 1 |
| 11 | Mmt_FEZ1 | gi|109109139| | Macaca mulatta | Eu., mt., vt., mam. | PREDICTED: zygin 1 |
| 12 | Cf_FEZ1 | gi|73954536| | Canis familiaris | Eu., mt., vt., mam. | PREDICTED: similar to FEZ1 |
| 13 | Pt_FEZ1 | gi|114641075| | Pan troglodytes | Eu., mt., vt., mam. | PREDICTED: zygin 1 isoform 1 |
| 14 | Rn_FEZ1 | gi|13994121| | Rattus norvegicus | Eu., mt., vt., mam. | FEZ1 |
| 15 | Hs_FEZ1 | gi|4826724| | Homo sapiens | Eu., mt., vt., mam. | FEZ1 |
| 16 | Mm_FEZ1 | gi|148693469| | Mus musculus | Eu., mt., vt., mam. | FEZ1 |
| 17 | Ec_FEZ2 | gi|194220815| | Equus caballus | Eu., mt., vt., mam. | PREDICTED: similar to zygin 2 |
| 18 | Tg_FEZ2 | gi|224047659| | Taeniopygia guttata | Eu., mt., vt., av. | PREDICTED: similar to zygin 2 |
| 19 | Gg_FEZ2 | gi|118101818| | Gallus gallus | Eu., mt., vt., av. | PREDICTED: hypothetical protein isoform 1 |
| 20 | Xt_FEZ2 | gi|62858603| | Xenopus tropicalis | Eu., mt., vt., amp. | Fez2 protein |
| 21 | Xl_FEZ2 | gi|67678016| | Xenopus laevis | Eu., mt., vt., amp. | LOC733280 protein |
| 22 | Xt_FEZ1 | gi|118405066| | Xenopus tropicalis | Eu., mt., vt., amp. | zygin 1 |
| 23 | Tn_FEZ1 | gi|47208647| | Tetraodon nigroviridis | Eu., mt., vt., acti. | unnamed protein product |
| 24 | Dr_FEZ2 | gi|116812571| | Danio rerio | Eu., mt., vt., acti. | zygin 2 |
| 25 | Dr_FEZ1 | gi|54261791| | Danio rerio | Eu., mt., vt., acti. | FEZ1 |
| 26 | Ss_FEZ1 | gi|213514730| | Salmo salar | Eu., mt., vt., acti. | FEZ1 |
| 27 | Ci_FEZ2 | gi|198425368| | Ciona intestinalis | Eu., mt., cph., tun | PREDICTED: similar to FEZ2 |
| 28 | Bf_FEZ2a | gi|219489321| | Branchiostoma floridae | Eu., mt., cph., bra | hypothetical protein BRAFLDRAFT_103854 |
| 29 | Bf_FEZ2b | gi|219411184| | Branchiostoma floridae | Eu., mt., cph., bra | hypothetical protein BRAFLDRAFT_202324 |
| 30 | Ce_UNC76 | gi|115534730| | Caenorhabditis elegans | Eu., mt., nem., chr. | UNC-76 |
| 31 | Bm_UNC76 | gi|170594585| | Brugia malayi | Eu., mt., nem., chr. | UNC-76 |
| 32 | Dpo_UNC76 | gi|198467847| | Drosophila pseudoobscura | Eu., mt., art., ins. | GA17820 |
| 33 | Ds_UNC76 | gi|195347821| | Drosophila sechellia | Eu., mt., art., ins. | GM19195 |
| 34 | Dy_UNC76 | gi|195477805| | Drosophila yakuba | Eu., mt., art., ins. | GE16233 |
| 35 | Dm_UNC76 | gi|18543271| | Drosophila melanogaster | Eu., mt., art., ins. | UNC-76 |
| 36 | Da_UNC76 | gi|194764284| | Drosophila ananassae | Eu., mt., art., ins. | GF21457 |
| 37 | Dw_UNC76 | gi|195447704| | Drosophila willistoni | Eu., mt., art., ins. | GK25735 |
| 38 | Dv_UNC76 | gi|195397079| | Drosophila virilis | Eu., mt., art., ins. | GJ16936 |
| 39 | Dc_UNC76 | gi|91081223| | Tribolium castaneum | Eu., mt., art., ins. | PREDICTED: similar to AGAP003014-PA |
| 40 | Dmj_UNC76 | gi|195129810| | Drosophila mojavensis | Eu., mt., art., ins. | GI15282 |
| 41 | Nv_UNC76 | gi|156548791| | Nasonia vitripennis | Eu., mt., art., ins. | PREDICTED: similar to ENSANGP00000018262 |
| 42 | Ag_UNC76 | gi|118781797| | Anopheles gambiae | Eu., mt., art., ins. | AGAP003014-PA |
| 43 | Pn_UNC76 | gi|242012918| | Pediculus humanus corporis | Eu., mt., art., ins. | Fasciculation and elongation protein zeta, putative |
| 44 | Am_UNC76 | gi|48103592| | Apis mellifera | Eu., mt., art., ins. | PREDICTED: similar to Unc-76 |
| 45 | Dg_UNC76 | gi|195046162| | Drosophila grimshawi | Eu., mt., art., ins. | GH24577 |
| 46 | Ap_UNC76 | gi|193586919| | Acyrthosiphon pisum | Eu., mt., art., ins. | PREDICTED: similar to AGAP003014-PA |
| 47 | Dp_UNC76 | gi|195170071| | Drosophila persimilis | Eu., mt., art., ins. | GL18226 |

acti. = Actinopterygii, amp. = Amphibia, art. = Arthropoda, cph = Cephalochordata, eu. = Eukaryota, ins. = Insecta, invt = Invertebrata, mam. = Mammalia, mt = Metazoa, nem = Nematoda, vt = Vertebrata, av. = Aves
